# Recent Advances in Layered Metal‐Oxide Cathodes for Application in Potassium‐Ion Batteries

**DOI:** 10.1002/advs.202105882

**Published:** 2022-04-27

**Authors:** Muthu Gnana Theresa Nathan, Hakgyoon Yu, Guk‐Tae Kim, Jin‐Hee Kim, Jung Sang Cho, Jeha Kim, Jae‐Kwang Kim

**Affiliations:** ^1^ Department of Energy Convergence Engineering Cheongju University Cheongju Chungbuk 28503 Republic of Korea; ^2^ Department of Biomedical Laboratory Science College of Health Science Cheongju University Cheongju Chungbuk 28503 Republic of Korea; ^3^ Department of Engineering Chemistry Chungbuk National University Chungbuk 28644 Republic of Korea

**Keywords:** cathode materials, intercalation chemistry, layered oxides, phase transitions, potassium‐ion batteries

## Abstract

To meet future energy demands, currently, dominant lithium‐ion batteries (LIBs) must be supported by abundant and cost‐effective alternative battery materials. Potassium‐ion batteries (KIBs) are promising alternatives to LIBs because KIB materials are abundant and because KIBs exhibit intercalation chemistry like LIBs and comparable energy densities. In pursuit of superior batteries, designing and developing highly efficient electrode materials are indispensable for meeting the requirements of large‐scale energy storage applications. Despite using graphite anodes in KIBs instead of in sodium‐ion batteries (NIBs), developing suitable KIB cathodes is extremely challenging and has attracted considerable research attention. Among the various cathode materials, layered metal oxides have attracted considerable interest owing to their tunable stoichiometry, high specific capacity, and structural stability. Therefore, the recent progress in layered metal‐oxide cathodes is comprehensively reviewed for application to KIBs and the fundamental material design, classification, phase transitions, preparation techniques, and corresponding electrochemical performance of KIBs are presented. Furthermore, the challenges and opportunities associated with developing layered oxide cathode materials are presented for practical application to KIBs.

## Graphical abstract

Layered oxide cathodes for potassium‐ion batteries have gained considerable attention in recent years. This review provides outline of recent research advances in layered cathodes including conceptual understanding, synthesis methods, structural characteristics, and electrochemical properties. In addition, some future research directions to further improve overall performance of layered oxide cathodes in potassium‐ion batteries are presented.

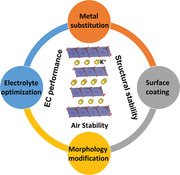

## 1 Introduction

**Figure 1:**
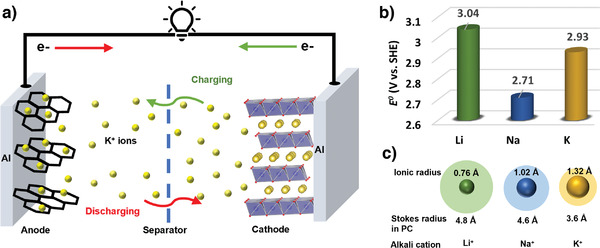
a) Schematic illustration of working mechanism of KIB, b) standard redox potential of various metal anodes, and c) comparison of Shannon's ionic radius and Stokes radius in propylene carbonate of Li^+^, Na^+^ and K^+^.

The ever‐growing energy demand and alarmingly increasing environmental pollution caused by the massive consumption of fossil fuels have driven the research community to focus on developing sustainable clean energy technologies.^[^ ^1^ ^]^ Toward this goal, energy conversion and storage devices are both equally important for overcoming the global energy crisis.^[^ ^2^, ^3^, ^4^, ^5^ ^]^ Among various energy storage systems, lithium‐ion batteries (LIBs) have been the predominant power sources for consumer electronics and smart wearable devices owing to their high energy density, long cycling life, and easy maintenance.^[^ ^6^, ^7^ ^]^ Recently, LIBs have been utilized in transportation fields to power (hybrid) electric vehicles, which minimize CO_2_ emissions and noise pollution.^[^ ^8^ ^]^ However, LIBs alone may not meet the future energy demands associated with the rapid growth of the electric vehicle market and stationary storage systems. Lithium is scarce and unevenly distributed in Earth's crust, which raises concerns about the soaring price of LIBs and the sustainability of lithium for meeting future energy demands.^[^ ^9^, ^10^ ^]^ Therefore, developing economical, high‐performance post‐LIBs is critical. Hence, sodium‐ion batteries (NIBs) and potassium‐ion batteries (KIBs) are promising alternatives or complements to LIBs because sodium and potassium are both abundant.^[^ ^9^, ^11^, ^12^ ^]^ Interestingly, like LIBs, NIBs and KIBs operate by a similar “rocking‐chair” mechanism. Therefore, extensive knowledge about LIBs enables the rapid development of NIBs and KIBs. A schematic illustrating the KIB operating principle is shown in **Figure** **1**a. Clearly, K^+^‐ions shuttle between the cathode and anode during charging and discharging.^[^ ^13^, ^14^ ^]^

Recently, KIBs have attracted considerable research interest because they have some advantages over NIBs and exhibit some properties comparable to those of LIBs. For instance, well‐established LIB graphite anodes are easily transferrable to KIBs because graphite can reversibly accommodate K^+^‐ions forming intercalation compounds such as KC_8_^[^ ^15^, ^16^ ^]^ in contrast to the formation of NC_70_, which limits Na^+^‐ion intercalation into graphite.^[^ ^17^ ^]^ The standard redox potentials of Li/Li^+^, Na/Na^+^, and K/K^+^ are −3.04, −2.71, and −2.93 V versus a standard hydrogen electrode (SHE), respectively (Figure 1b). The standard redox potential of K/K^+^ is comparable to that of Li/Li^+^ in aqueous electrolytes. Furthermore, the K/K^+^ redox couple exhibits even lower standard redox potentials than Li/Li^+^ in nonaqueous electrolytes such as propylene carbonate (PC) (−0.09 V vs Li/Li^+^) and a mixture consisting of ethylene carbonate and diethyl carbonate (EC:DEC) (−0.12 V vs Li/Li^+^).^[^ ^18^, ^19^ ^]^ The low K/K^+^ redox potential leads to a wider potential window for operating KIBs and will eventually achieve high‐energy‐density batteries. Alkali metal ion (e.g., Li^+^, Na^+^, and K^+^) transport properties influence the rate performance of this battery class. As shown in Figure 1c, although K^+^ (1.38 Å) ions are larger than Na^+^ (1.02 Å) and Li^+^ (0.76 Å) ones, K^+^ ions exhibit lower charge density and the lowest solvation and desolvation energies among the alkali metal ions, which facilitates rapid desolvation at the electrode–electrolyte interface. Furthermore, because K^+^‐ions weakly interact with solvent molecules, the smaller K^+^‐ion Stokes radius (Figure 1c) results in higher ionic conductivities and transference numbers.^[^ ^20^, ^21^ ^]^ Another advantage of KIBs over LIBs is that inexpensive aluminum current collectors can be used on both the cathode and anode sides because unlike lithium, potassium does not alloy with aluminum—which reduces the KIB cost and weight.^[^ ^22^, ^23^ ^]^ Regarding safety, NIBs exhibit an Na^+^‐ion insertion potential of 0.05 V versus Na/Na^+^ for a hard carbon anode, which is too close to the sodium metal plating potential, implying that dendrites will form in NIBs cycled at high current rates. In contrast, KIBs exhibit an average K^+^‐ion intercalation potential of 0.2 V versus K/K^+^ for most carbon anodes, which is well above the potassium metal plating potential, suggesting that KIBs are safer than NIBs.^[^ ^24^, ^25^ ^]^ However, although all these advantages make KIBs a promising alternative to LIBs, potassium metal must be handled with the utmost care when fabricating half cells because potassium metal is highly flammable.

The reversible intercalation of K^+^ ions into graphite anodes has shifted the research focus to designing and exploring suitable cathode materials for practical application to KIBs. Obviously, the cathode is the key component determining the KIB electrochemical characteristics, energy density, and cost. Recently, extensive research efforts have led to the development of various cathode materials such as Prussian blue analogs,^[^ ^26^, ^27^ ^]^ layered metal oxides,^[^ ^28^, ^29^ ^]^ polyanionic frameworks,^[^ ^30^, ^31^ ^]^ and organic compounds.^[^ ^32^, ^33^ ^]^ The application of layered metal‐oxide cathodes (such as LiCoO_2_) to commercial LIBs has attracted considerable interest in storing K^+^ ions in layered metal‐oxide cathodes for application to KIBs because the cathodes exhibit high capacity, large K^+^‐ion diffusion paths, and scalable synthesis. Recently, various groups have published reviews of KIB electrode materials and electrolytes.^[^ ^9^, ^14^, ^18^, ^19^, ^20^, ^21^, ^23^, ^34^, ^35^, ^36^, ^37^, ^38^ ^]^ From this perspective, a comprehensive review of cathode material advantages and challenges is critical to further develop KIBs.

Therefore, we report recent research progress on layered metal oxide cathodes for application to KIBs. First, we introduce the KIB charge storage mechanism and the advantages of KIBs over NIBs and LIBs. Then, the layered metal oxide structural classification and phase transitions are discussed. Recent works related to layered metal oxide cathodes for application to KIBs are summarized based on the number of transition metals used by focusing on the structural transformations and electrochemical performances of half‐ and full‐cell configurations. Finally, some strategies are proposed to suppress irreversible phase transitions and enhance the overall performance for future KIB development.

## 2 Structural Classification of Layered Transition‐Metal Oxides

**Figure 2:**
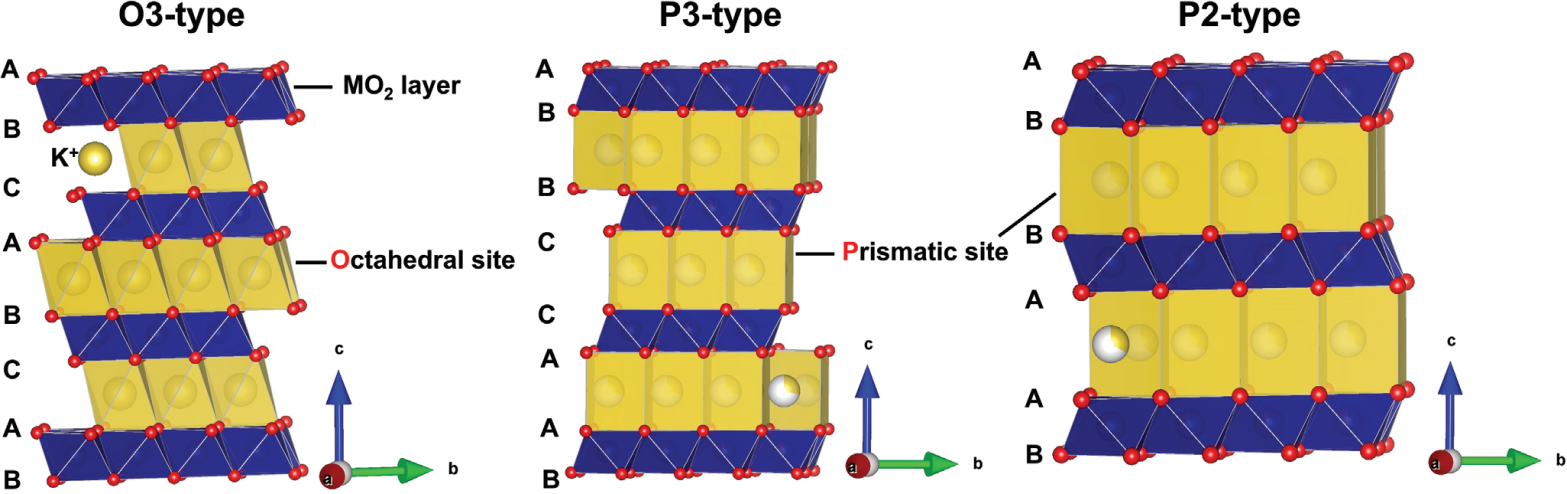
Crystal structures of O3‐, P3‐, and P2‐type layered metal oxides.

Layered transition‐metal oxides can be represented by the formula A*_x_* MO_2_ (0 < *x* < 1), where A represents alkali metal ions (e.g., Li^+^, Na^+^, and K^+^), and M can be one or more transition‐metal ions in various oxidation states. Typically, layered K*_x_* MO_2_ compounds are formed with alternately stacked edge‐sharing MO_6_ octahedral layers and K^+^‐ion layers. P2‐, P3‐, and O3‐type layered K*_x_* MO_2_ can be synthesized based on the surrounding K^+^‐ion environment and the number of unique oxide layer stacking sequences.^[^ ^39^ ^]^ P and O indicate whether K^+^ ions are in a prismatic or an octahedral coordination environment, respectively. Numbers 2 and 3 indicate the number of oxide layers in a single unit cell. **Figure** **2** shows a schematic illustrating the O3, P3, and P2 crystal structures. In O3 crystals, all the K^+^‐ions occupy octahedral sites, and oxide layer stacking follows the AB–CA–BC pattern. At high K^+^ concentrations (e.g., *x* = 1), strong electrostatic K^+^–K^+^ repulsion destabilizes K*_x_* MO_2_ layered compounds. O3‐KCrO_2_ is the only electrochemically active K*_x_* MO_2_ layered compound that forms O3 crystals.^[^ ^40^ ^]^ In P3 crystals, oxide layer stacking follows the AB–BC–CA pattern, and K^+^ ions are at prismatic sites. P2 compounds are formed by AB–BA oxide layer stacking. In P2 crystals, K^+^ ions occupy distinct edge‐ or face‐sharing prismatic sites.^[^ ^41^ ^]^ A prime symbol (ʹ) is used to specify the in‐plane distortion of hexagonal crystal lattices such as monoclinic Pʹ3‐K_0.8_CrO_2_.^[^ ^42^ ^]^

The reaction temperature is critical for obtaining different crystals. Most P2 compounds stabilize at higher temperatures than P3 ones. For instance, P3‐K*_x_* CoO_2_ and P2‐K*_x_* CoO_2_ are synthesized at 400 ° C and 600 ° C, respectively.^[^ ^43^ ^]^ The P3–P2 phase transformation occurs by breaking M—O bonds at high temperatures. Layered compound electrochemical behaviors are influenced by both the initial pristine compound K^+^‐ion content and structural stability. During K^+^‐ion extraction/insertion, O3 transitions to other phases because the MO_2_‐layer glides without breaking any M—O bonds. When a trace of K^+^‐ions is extracted, layer gliding changes the O3 oxide stacking pattern to the P3 one, which may be because larger K^+^‐ions prefer to occupy energetically favorable prismatic sites rather than smaller octahedral ones.^[^ ^40^ ^]^ Neither O3 nor P3 materials can electrochemically transform into P2 ones because M—O bonds cannot break to form P2 materials during charging and discharging. However, P2 materials transition to O2 ones when the maximum K^+^‐ion concentration is extracted.^[^ ^41^, ^44^ ^]^ Therefore, owing to fewer phase transitions, P compounds are structurally more stable than O layered compounds.

## 3 Layered Metal Oxide Cathodes for KIBs

### 3.1 Single Metal Oxides

#### 3.1.1 Manganese‐Based Electrodes

**Figure 3:**
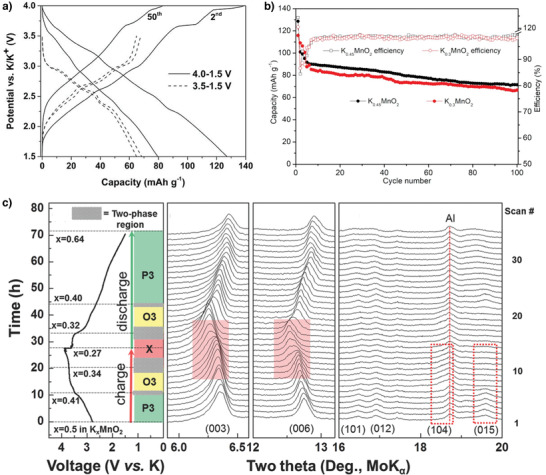
a) Potential profiles of the 2^nd^ and 50^th^ cycle at 0.1 C in the voltage range 3.5–1.5 and 4.0–1.5 V. Reproduced with permission.^[^ ^45^ ^]^ Copyright 2016, Electrochemical Society. b) Cycling performance of P2‐K_0.3_MnO_2_ and P3‐K_0.45_MnO_2_ at 20 mA g^−1^. Reproduced with permission.^[^ ^46^ ^]^ Copyright 2019, Elsevier. c) Structural changes of P3‐K_0.5_MnO_2_ during charge and discharge. Reproduced with permission.^[^ ^47^ ^]^ Copyright 2017, Wiley.

Vaalma et al. demonstrated the first‐ever nonaqueous KIB utilizing layered K_0.3_MnO_2_ as cathode, which exhibited an initial discharge capacity of 70 mAh g^−1^ and a reasonable capacity retention of 57% over 685 cycles at 27.9 mA g^−1^ in the potential window 1.5–3.5 V versus K/K^+^ (**Figure** **3**a).^[^ ^45^ ^]^ Nevertheless, K_0.3_MnO_2_ exhibited considerable capacity fading under the higher cutoff condition (1.5–4.0 V) possibly because of irreversible phase transitions at higher potentials. Furthermore, a KIB full cell was constructed as a proof‐of‐concept model, with K_0.3_MnO_2_ as the cathode and a hard carbon/carbon black composite as the anode, which encouraged researchers to develop different KIB electrode materials.^[^ ^45^ ^]^ The number of K^+^‐ions in layered oxides plays a crucial role in obtaining different structures, morphologies, and electrochemical performances. Liu et al. synthesized P2‐K_0.3_MnO_2_ and P3‐K_0.45_MnO_2_ layered oxides by varying the K^+^‐ion concentration under the same experimental conditions.^[^ ^46^ ^]^ Reportedly, the higher‐K^+^ P3‐K_0.45_MnO_2_ displayed smaller particles and slightly better cycling stability and rate performance than P2‐K_0.3_MnO_2_ (Figure 3b). In a wider potential window of 1.5–4.0 V versus K/K^+^, P3‐K_0.45_MnO_2_ delivered a specific capacity of 128.6 mAh g^−1^ at 20 mA g^−1^ and better rate performance with a specific capacity of 51.2 mAh g^−1^, even at a current density of 200 mA g^−1^.^[^ ^46^ ^]^

Kim et al. investigated the P3‐K_0.5_MnO_2_ electrochemical performance and structural changes during reversible K^+^‐ion deintercalation/intercalation.^[^ ^47^ ^]^ The in situ XRD patterns generated during charging/discharging and corresponding computations elucidated the P3‐K_0.5_MnO_2_ K^+^‐ion storage mechanism and how the K^+^‐content influenced the phase changes. As shown in the in situ XRD patterns (Figure 3c), P3‐K_0.5_MnO_2_ reversibly transitions among P3, O3, and *X* during K^+^‐ion extraction and reinsertion. P3‐K_0.5_MnO_2_ delivered a specific capacity of 106 mAh g^−1^ when cycled between 1.5 and 3.9 V versus K/K^+^, and further increasing the potential to 4.2 V resulted in considerable stacking‐fault‐induced capacity fading when more K^+^‐ions were extracted at high potentials; thus, the voltage range must be appropriately tuned to optimize durable performance.^[^ ^47^ ^]^ Structural morphology and particle size also play important roles in improving electrochemical performance. For instance, Peng et al. prepared P3‐K_0.5_MnO_2_ hollow submicrospheres (HSMSs) using two‐step self‐templating.^[^ ^48^ ^]^ When tested as a KIB cathode material in the range 1.5–3.9 V versus K/K^+^, the P3‐K_0.5_MnO_2_ HSMSs demonstrated a capacity of 104 mAh g^−1^ at 10 mA g^−1^ and excellent capacity retention of 89.1% over 400 cycles at 200 mA g^−1^. The enhanced electrochemical performance was ascribed to the synergy between the particle size and the hollow spherical morphology. The full cell assembled with a graphite anode and the P3‐K_0.5_MnO_2_ HSMS cathode delivered an energy density of 100.7 Wh kg^−1^ at an average output of 2.09 V, which highlights the HSMS feasibility for practical application to KIBs.^[^ ^48^ ^]^

**Figure 4:**
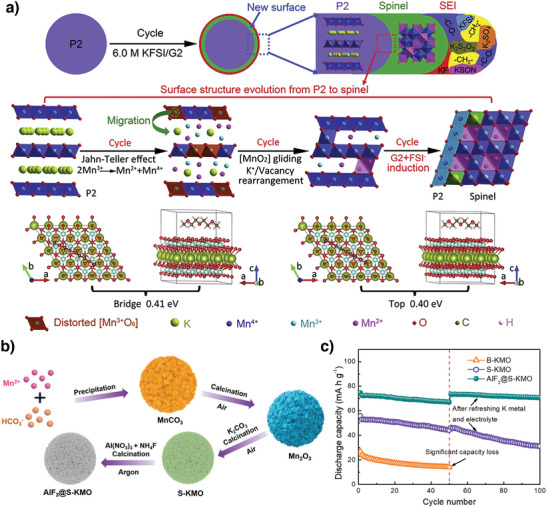
a) Formation mechanism of the dual interphase layers composed of a SEI layer and spinel interlayer on P2‐KMO during cycles. Reproduced with permission.^[^ ^53^ ^]^ Copyright 2019, Elsevier. b) Schematic illustration of the synthesis process of AlF_3_@S‐KMO. c) Cycling performance at 50 mA g^−1^ before and after refreshing the potassium metal anodes and electrolyte. Reproduced with permission.^[^ ^54^ ^]^ Copyright 2019, Wiley.

The major issues with manganese‐based layered cathodes are Jahn–Teller (J–T) active Mn^3+^ ions and the associated disproportionation reaction (2Mn^3+^ → Mn^2+^ + Mn^4+^), which generate an asymmetric cathode structure and lead to Mn^2+^‐ion dissolution into the electrolyte during cycling.^[^ ^49^, ^50^, ^51^, ^52^ ^]^ To circumvent these issues and stabilize Mn‐based cathode performance, different strategies have been employed. Notably, Lei et al. reported the in situ formation of a dual interface on an Mn‐based P2‐K_0.67_MnO_2_ cathode consisting of an inactive K‐poor spinel interlayer and a stable solid–electrolyte interface (SEI) film (**Figure** **4**a). The dual interface layers accommodated J‐T distortion, alleviated Mn dissolution, and improved the K^+^‐ion diffusion kinetics, which resulted in good rate performance, a small volumetric change of 9.9%, stable operation for 300 cycles, and a capacity retention of 90.5% at 50 mA g^−1^.^[^ ^53^ ^]^

Zhao et al. coated the surface of K_1.39_Mn_3_O_6_ microspheres with AlF_3_ to enhance their electrochemical performance (Figure 4b).^[^ ^54^ ^]^ The AlF_3_‐coated K_1.39_Mn_3_O_6_ delivered a specific capacity of 110 mAh g^−1^ at 10 mA g^−1^ and outstanding cycling stability with a capacity retention of 94.9% over 100 cycles, which was far superior to the electrochemical performances of both the noncoated microspheres and the bulk counterpart (Figure 4c).^[^ ^54^ ^]^ Therefore, surface modification is an efficient method of mitigating unwanted parasitic reactions and stabilizing cathode interfaces.^[^ ^54^ ^]^

#### 3.1.2 Cobalt‐Based Electrodes

**Figure 5:**
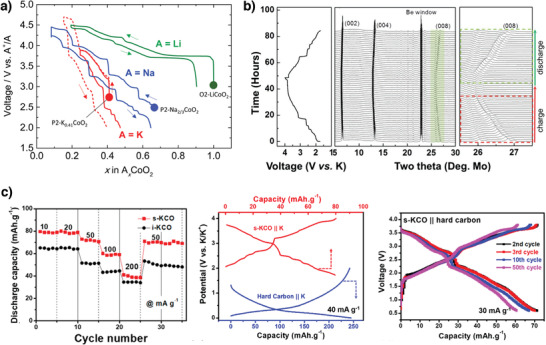
a) Voltage curves of A//AxCoO_2_ (A = Li, Na, and K). Reproduced with permission.^[^ ^43^ ^]^ Copyright 2017, Royal Society of Chemistry. b) Typical charge/discharge profile at a current rate of 2 mA g^−1^ and in situ XRD patterns of P2‐K_0.6_CoO_2_. Reproduced with permission.^[^ ^56^ ^]^ Copyright 2017, Wiley. c) Rate capability of s‐KCO and i‐KCO at different current rates; Typical charge‐discharge curves of s‐KCO//K and hard carbon//K in a half‐cell and s‐KCO//hard carbon full‐cell configurations. Reproduced with permission.^[^ ^57^ ^]^ Copyright 2018, American Chemical Society.

Although Delmas et al. synthesized and structurally characterized K*_x_* CoO_2_ crystals in 1975,^[^ ^55^ ^]^ K^+^‐ion deintercalation/intercalation was not studied for K*_x_* CoO_2_ compounds until 2017 by Hironaka et al.^[^ ^43^ ^]^ Because the synthesized P2‐K_0.41_CoO_2_ and P3‐K_2/3_CoO_2_ were moisture sensitive, they had to be handled in an argon atmosphere. Both P2‐K_0.41_CoO_2_ and P3‐K_2/3_CoO_2_ delivered similar specific capacities of ≈60 mAh g^−1^ in the potential window 2.0–3.9 V versus K/K^+^. The multistep voltage profile originated from strong K^+^/vacancy ordering, which is supported by the operando XRD results. Compared to O2‐LiCoO_2_, P2‐Na_2/3_CoO_2_ and P2‐K_0.41_CoO_2_ both displayed a steep voltage drop (**Figure** **5**a), which mainly depended on the ionic radius interslab distance and determined the alkali‐metal ion battery working voltage.^[^ ^43^ ^]^ P2‐K_0.6_CoO_2_ synthesized with a higher K^+^‐ion content delivered a specific capacity of 80 mAh g^−1^ at 2 mA g^−1^ and average output of 2.7 V versus K/K^+^.^[^ ^56^ ^]^ P2‐K_0.6_CoO_2_ maintained the P2 structure when the K^+^‐ion content was varied between 0.33 and 0.68, thereby implying a reversible topotactic reaction (Figure 5b); however, P2‐K_0.6_CoO_2_ exhibited poor cycling stability likely due to side reactions with the electrolyte.

In another study, P2‐K_0.6_CoO_2_ (s‐KCO) microspheres were synthesized using self‐templating.^[^ ^57^ ^]^ The s‐KCO electrode demonstrated a high specific capacity of 82 mAh g^−1^ at 10 mA g^−1^, high‐rate capability, and excellent cycling stability with 87% capacity retention over 300 cycles at 40 mA g^−1^ (Figure 5c). The hierarchical microsphere structure provided fast K^+^‐ion and electron transport pathways and minimized the contact area between the electroactive materials and the electrolyte, thus reducing undesirable side reactions and enhancing K^+^‐ion storage in P2‐K_0.6_CoO_2_. Furthermore, the s‐KCO//hard carbon full cell exhibited a high capacity of 71 mAh g^−1^ at 30 mA g^−1^ and excellent capacity retention (>80%) after 100 cycles.^[^ ^57^ ^]^

#### 3.1.3 Chromium‐Based Electrodes

**Figure 6:**
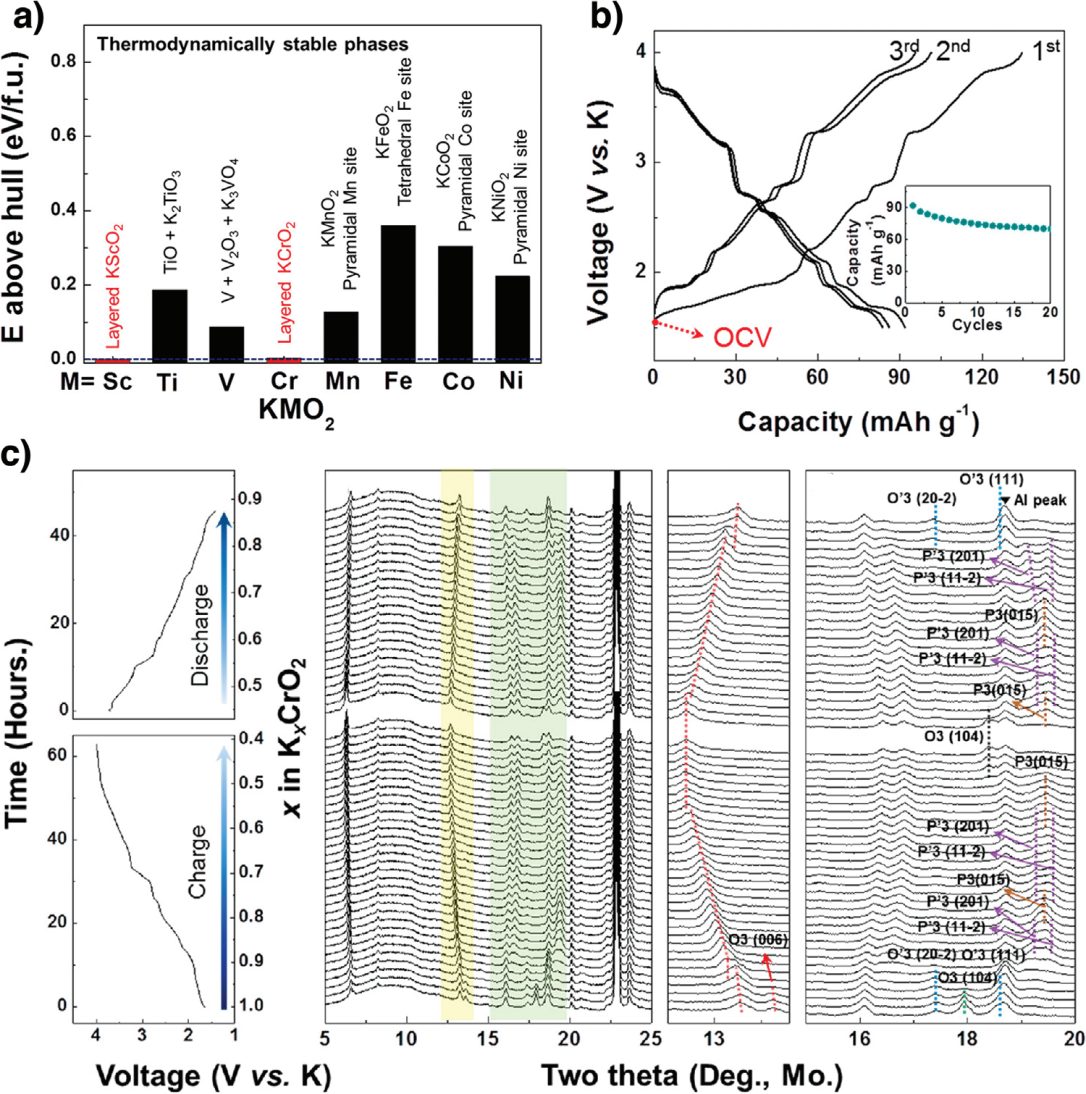
a) Thermodynamic stability of layered KMO_2_ compounds. b) Typical voltage‐capacity curves at a current rate of 5 mA g^−1^ and c) in situ XRD patterns of O3‐KCrO_2_. Reproduced with permission.^[^ ^40^ ^]^ Copyright 2018, American Chemical Society.

Most layered K^+^‐ion compounds prepared are having lower K^+^ concentrations than sodium and lithium analogs^[^ ^43^, ^58^, ^59^, ^60^ ^]^ because strong K^+^–K^+^ repulsion cannot accommodate all the K^+^ ions in the K^+^‐ion layer, thus generating K^+^‐deficient compounds (K*_x_* MO_2_; *x* ≤ 0.7). Computational studies have indicated that only KScO_2_ and KCrO_2_ are thermodynamically stable layered compounds (**Figure** **6**a). Kim et al. prepared stoichiometrically layered O3‐KCrO_2_ and investigated its electrochemical performance as a KIB cathode.^[^ ^40^ ^]^ The layered KCrO_2_ was stabilized owing to the unusual Cr^3+^‐ligand‐field preference for octahedral sites, which compensated for the K^+^–K^+^‐repulsion‐induced energy penalty. O3‐KCrO_2_ delivered a discharge capacity of 92 mAh g^−1^ at 5 mA g^−1^ and exhibited a multistep voltage profile (Figure 6b). During charging, the O3‐KCrO_2_ cathode reversibly transitioned among O3–Oʹ3–Pʹ3–P3–Pʹ3–P3–O3 (Figure 6c). These phase transitions are more complex than those observed for O3‐NaCrO_2_, which is attributed to strong K^+^–K^+^ interactions. Moreover, the incomplete recovery of K^+^ ions—even at the end of discharging—shows that the Oʹ3 structure is not converted into the O3 one, which is likely owing to the sluggish K^+^‐ion kinetics when *x* ≈ 1 in K*_x_* CrO_2_. The full cell constructed using the O3‐KCrO_2_ cathode and a graphite anode exhibited a capacity of ≈82 mAh g^−1^ at 5 mA g^−1^.^[^ ^40^ ^]^ This work provides insight into the design of stoichiometrically layered compounds for application to KIBs. Naveen et al. synthesized layered O3‐KCrS_2_ with a stoichiometric amount of K^+^. O3‐KCrS_2_ exhibited high electrochemical reversibility between P3‐K_0.39_CrS_2_ and O3‐K_0.80_CrS_2_ because the material mostly retains the P3 structure

during K^+^ deintercalation/intercalation, thereby enabling fast K^+^‐ion diffusion through larger prismatic sites. The O3‐KCrS_2_ cathode delivered a specific capacity of 71 mAh g^−1^ and excellent cycling stability with ≈90% capacity retention over 1000 cycles owing to the soft sulfide framework, which buffered the K^+^‐deinsertion/insertion‐induced stress.^[^ ^61^ ^]^

Hwang et al. prepared another Cr‐based cathode, P3‐K_0.69_CrO_2_, from O3‐NaCrO_2_ through electrochemical ion exchange, which is an efficient process when synthesizing layered compounds is difficult using the conventional solid‐state method.^[^ ^62^, ^63^, ^64^, ^65^ ^]^ The P3‐K_0.69_CrO_2_ cathode exhibited a discharge capacity of 100 mAh g^−1^ at 10 mA g^−1^ in the range 1.5–3.8 V versus K/K^+^. Although this cathode exhibited staircase‐like voltage profiles, it reversibly transitioned between P3 and P″3 and exhibited outstanding long‐term cyclability at 100 mA g^−1^ with 65% capacity retention over 1000 cycles.^[^ ^66^ ^]^ Further studies are required to optimize the electrolyte and reduce the slow ion exchange to improve the potential for practical applications. Naveen et al. developed a facile method of synthesizing Pʹ3‐K0.8CrO_2_.^[^ ^42^ ^]^ The Pʹ3‐K_0.8_CrO_2_ cathode displayed different O3‐KCrO_2_ phase transitions, resulting in different electrochemical performances. The absence of the O3 phase and persistence of the Pʹ3 ones in most redox states contributed to limited volumetric changes (Δ*V* = 1.08%) and rapid K^+^‐ion diffusion, which eventually resulted in improved cyclability and 99% capacity retention at 218 mA g^−1^ after 300 cycles.^[^ ^42^ ^]^

#### 3.1.4 Vanadium‐Based Electrodes

**Figure 7:**
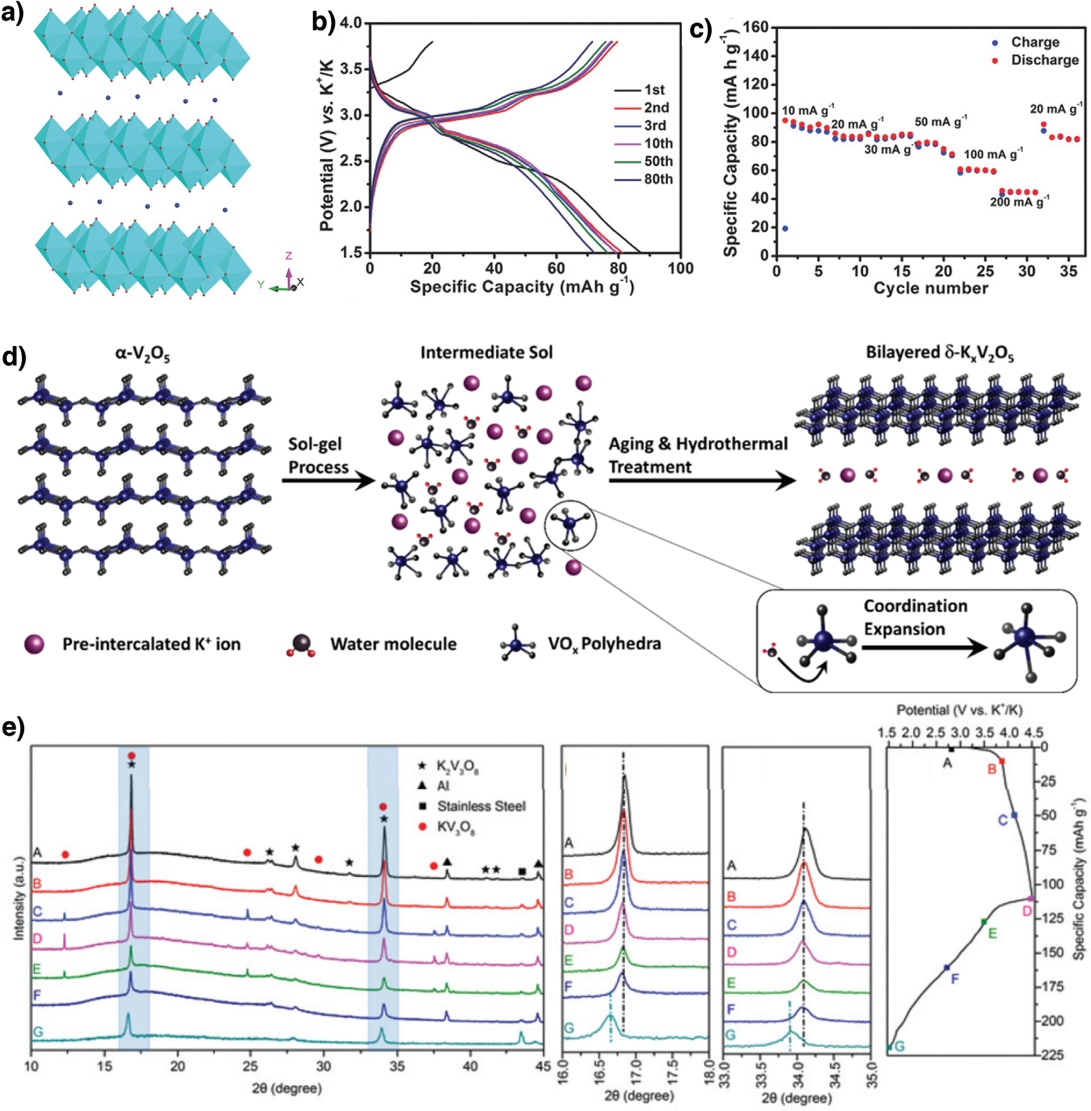
a) Crystal structure of K_0.5_V_2_O_5,_ b) galvanostatic charge/discharge voltage profiles, c) rate capability of K_0.5_V_2_O_5_. Reproduced with permission.^[^ ^71^ ^]^ Copyright 2018, Wiley. d) Schematic illustration of chemical pre‐intercalation synthesis approach. Reproduced with permission.^[^ ^72^ ^]^ Copyright 2018, American Chemical Society. e) Ex situ XRD patterns of the K_2_V_3_O_8_ electrode at various charge/discharge states at 10 mA g^−1^. Reproduced with permission.^[^ ^73^ ^]^ Copyright 2019, Royal Society of Chemistry.

Vanadium oxides have been investigated as cathode and anode materials for application to rechargeable batteries owing to their versatile structures, multiple vanadium valence states, high specific capacities, and high electrochemical reactivity.^[^ ^67^, ^68^, ^69^, ^70^ ^]^ K_0.5_V_2_O_5_ exhibits a layered structure formed by edge‐sharing octahedral VO_6_ and K^+^ ions sandwiched between the layers (**Figure** **7**a).^[^ ^71^ ^]^ The K_0.5_V_2_O_5_ cathode delivered a specific capacity of 90 mAh g^−1^ at 10 mA g^−1^ in the range 1.5–3.8 V versus K/K^+^. To prepare a full cell using carbon‐based anodes, a suitable cathode must exhibit numerous extractable K^+^ ions because the cathode serves as a reservoir for reversible K^+^ ions. Although only a trace of K^+^ ions was extracted from K_0.5_V_2_O_5_ during the initial charging (Figure 7b), this problem was solved by prepotassiation.^[^ ^71^ ^]^ Clites et al. prepared K^+^‐preintercalated bilayered K*_x_* V_2_O_5_·*n*H_2_O by the sol–gel method.^[^ ^72^ ^]^ During synthesis, K^+^ ions and water molecules are trapped between growing vanadium oxide bilayers to form *δ*‐K*_x_* V_2_O_5_·*n*H_2_O (Figure 7d). The *δ*‐K*_x_* V_2_O_5_·*n*H_2_O cathode demonstrated a high initial capacity of 226 mAh g^−1^ at 20 mA g^−1^. K^+^‐ion extraction and insertion were accompanied by a reversible change between V^5+^ and V^3+^ involving two electrons. The enhanced *δ*‐K*_x_* V_2_O_5_·*n*H_2_O performance is attributed to well‐defined sites for electrochemically cycled K^+^ ions and the large interlayer spacing achieved through chemical preintercalation.^[^ ^72^ ^]^

Yang et al. hydrothermally synthesized K_2_V_3_O_8_, and the cathode delivered a discharge capacity of 107.8 mAh g^−1^ at 10 mA g^−1^.^[^ ^73^ ^]^ The use of a highly concentrated electrolyte [7 m potassium bis(fluorosulfonyl)imide (KFSI) dissolved in EC:DEC] supported K_2_V_3_O_8_ cycling in a wide potential range 4.5–1.5 V versus K/K^+^, and the cathode discharged an average of 2.7 V. However, the cathode exhibited poor cycling stability with only 73% capacity retention after 50 cycles. Ex situ XRD (Figure 7e) revealed that the capacity degraded because K_2_V_3_O_8_ undergoes nontopotactic K^+^‐ion extraction/insertion and reversibly changes between K_2_V_3_O_8_ and K_2_V_3_O_8_ accompanied by huge volumetric changes (Δ*V* ≈ 23.4%), which caused the electrode materials to mechanically fail.^[^ ^73^ ^]^ In the context of layered compounds prepared with high initial K^+^‐ion contents, Zhang et al. synthesized K_0.83_V_2_O_5_.^[^ ^74^ ^]^ The cathode delivered an initial charge capacity of 86 mAh g^−1^ and a reversible capacity of 90 mAh g^−1^ at 10 mA g^−1^. Furthermore, it exhibited cycling stability with 86% capacity retention over 200 cycles. The full cell fabricated using the K_0.83_V_2_O_5_ cathode and a graphite anode demonstrated an energy density of 136 Wh kg^−1^ with an average output of 2.4 V. This study provides some insights for designing high‐K^+^‐content layered cathode materials for application to emerging practical KIBs.^[^ ^74^ ^]^

In short, the layered single metal oxide cathodes demonstrated reversible K^+^ deintercalation/intercalation suitable for K^+^ storage in KIBs. However, these single metal compounds suffer from poor air stability, complex phase transitions, slope voltage, capacity fading, and insufficient cycling stability. Therefore, modifying the structure and composition by substituting suitable metals could alter the crystal lattice parameters and thus leading to improved structural stability and electrochemical performance.

### 3.2 Dual Metal‐Based Electrodes

Although researchers initially focused on single metal oxide‐based electrodes to explore layered cathodes for application to KIBs, the shortcomings of single metal oxide‐based cathodes such as low capacity, structural instability, multistep voltages, rapid capacity decay, and low average voltages must be overcome to design efficient and durable cathodes for application to practical KIBs. Although Mn‐based layered compounds appear to be promising materials because of their reasonable capacities, low cost, and environmentally benign manganese, Mn^3+^‐induced J‐T distortion and related structural changes lead to poor electrochemical performance.^[^ ^75^, ^76^, ^77^ ^]^ To circumvent these issues, many researchers have doped Mn with other metals and eventually enhanced electrochemical performance.

#### 3.2.1 Mn/Fe‐Based Electrodes

**Figure 8:**
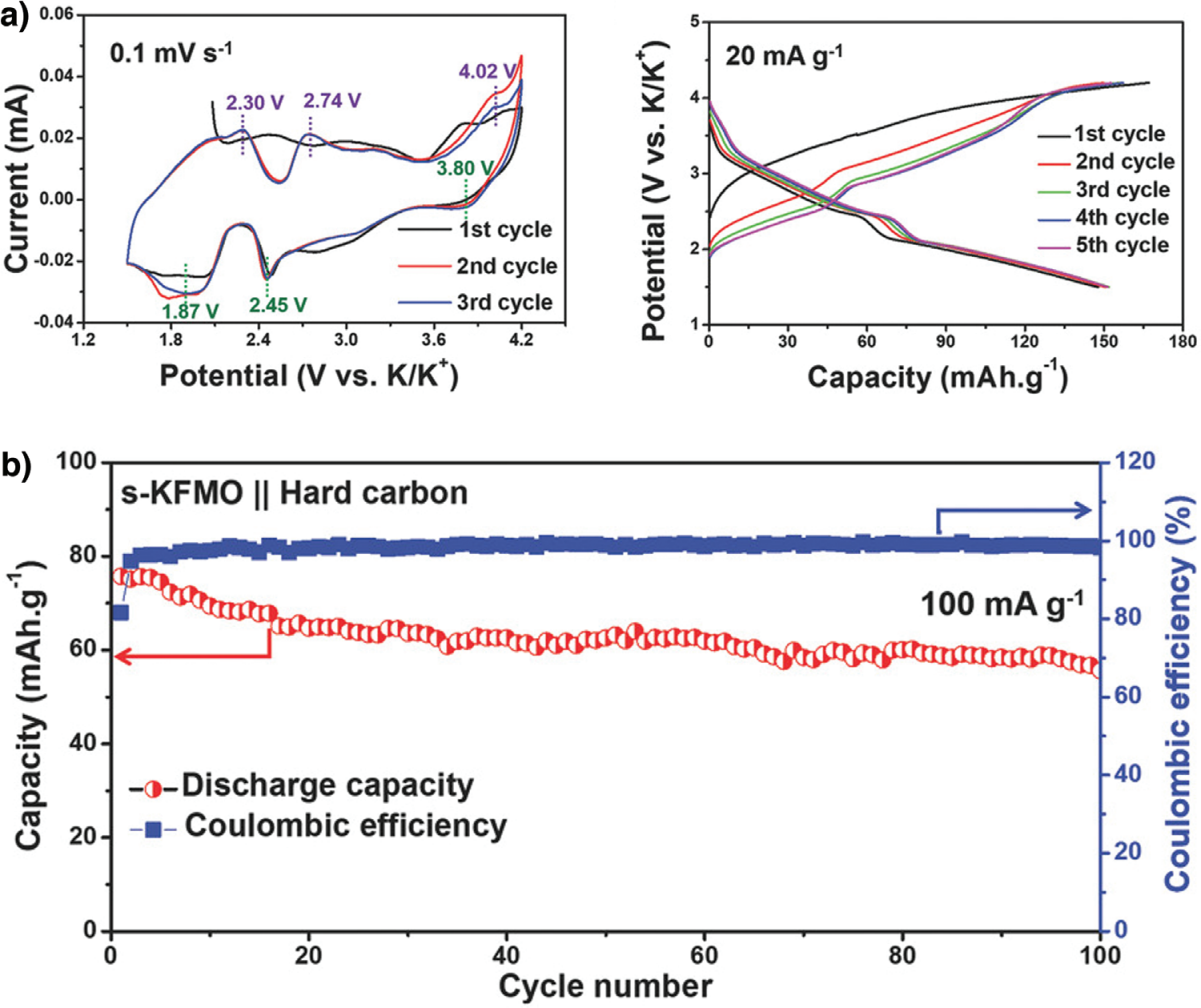
a) CV curves of s‐KFMO electrode at a scanning rate of 0.1 mV s^−1^ and charge/discharge curves of s‐KFMO cathode at 20 mA g^−1^, b) cycling performance of s‐KFMO//hard carbon full cell. Reproduced with permission.^[^ ^44^ ^]^ Copyright 2018, Wiley.

Designing electrodes using low‐cost abundant materials is attractive because it will reduce the overall battery cost. Wang et al. reported the first‐ever abundant Fe/Mn‐based K_0.7_Fe_0.5_Mn_0.5_O_2_ layered oxide cathode nanowires, which delivered a high discharge capacity of 178 mAh g^−1^ at 20 mA g^−1^.^[^ ^78^ ^]^ The unique interconnected K_0.7_Fe_0.5_Mn_0.5_O_2_ nanowire morphology resulted in superior cycling stability with 87% capacity retention at 500 mA g^−1^ after 200 cycles. Furthermore, the K_0.7_Fe_0.5_Mn_0.5_O_2_//soft carbon full cell exhibited a capacity of 119 mAh g^−1^ at 20 mA g^−1^, thereby demonstrating its suitability as a cathode material for application to KIBs.^[^ ^78^ ^]^ P2‐K_0.65_Fe_0.5_Mn_0.5_O_2_ microspheres (s‐KFMO) were solvothermally prepared by Deng et al. The cathode material exhibited a highly reversible K^+^‐ion storage capacity of 151 mAh g^−1^ at 20 mA g^−1^. The CV curves (**Figure** **8**a) show that the charge compensation mechanism involves both low‐spin Mn^3+^/Mn^4+^ and high‐spin Fe^3+^/Fe^4+^ redox couples above 3.6 V. A full cell constructed using an s‐KFMO cathode and a hard carbon anode demonstrated long‐term cycle stability with 80% capacity retention over 100 cycles (Figure 8b). The secondary microsphere structure decreases the contact area between the active materials and the electrolyte, thus minimizing unwanted side reactions and leading to high Coulombic efficiency.^[^ ^44^ ^]^

Liu et al. synthesized a series of Fe‐doped K_0.45_Mn_1−_ *_x_* Fe*_x_* O_2_ (*x* ≤ 0.5) to study the influence of Fe substitution on the electrochemical performance. Among the synthesized materials, K_0.45_Mn_0.8_Fe_0.2_O_2_ delivered a discharge capacity of 106.2 mAh g^−1^ at 20 mA g^−1^ and the best cycling and rate performances. Excess Fe doping (*x* > 0.3) decreases the electrode capacity because most electroactive Mn ions are replaced by Fe ions, which do not participate in the redox reaction. Optimizing the Fe doping content could reduce the cathode material polarization and enhance the cathode structural stability.^[^ ^79^ ^]^ Masese et al. developed a unique K_0.4_Fe_0.5_Mn_0.5_O_2_ layered oxide cathode comprising trivalent Fe^3+^ and tetravalent Mn^4+^, unlike other Fe/Mn‐based layered oxides comprising both trivalent Fe^3+^ and Mn^3+^.^[^ ^80^ ^]^ This cathode material delivered a reversible specific capacity of 120 mAh g^−1^ and an average discharge of 2.8 V. X‐ray absorption near‐edge structure (XANES) spectra revealed that charge compensation involved the cumulative participation of transition‐metal cations and oxygen anion redox reactions during K^+^‐ion extraction and reinsertion.^[^ ^80^ ^]^ Because such an oxygen anion redox mechanism is rarely observed for K^+^‐ion layered oxides, more investigations are required to elucidate anion redox chemistry.

In short, the Fe metal doping in K*_x_* MnO_2_ system enhanced specific capacity and an average voltage corresponding to Fe^3+/4+^ redox couple. From the viewpoint of commercial batteries, the Mn/Fe‐based electrodes may be a more suitable choice as these elements are earth abundant, low‐cost, and nontoxic. However, the J–T effect of Mn^3+^ and Fe^4+^ ions must be tackled, and thus further studies are necessary to optimize these materials for achieving durable battery performance.

#### 3.2.2 Mn/Co‐Based Electrodes

**Figure 9:**
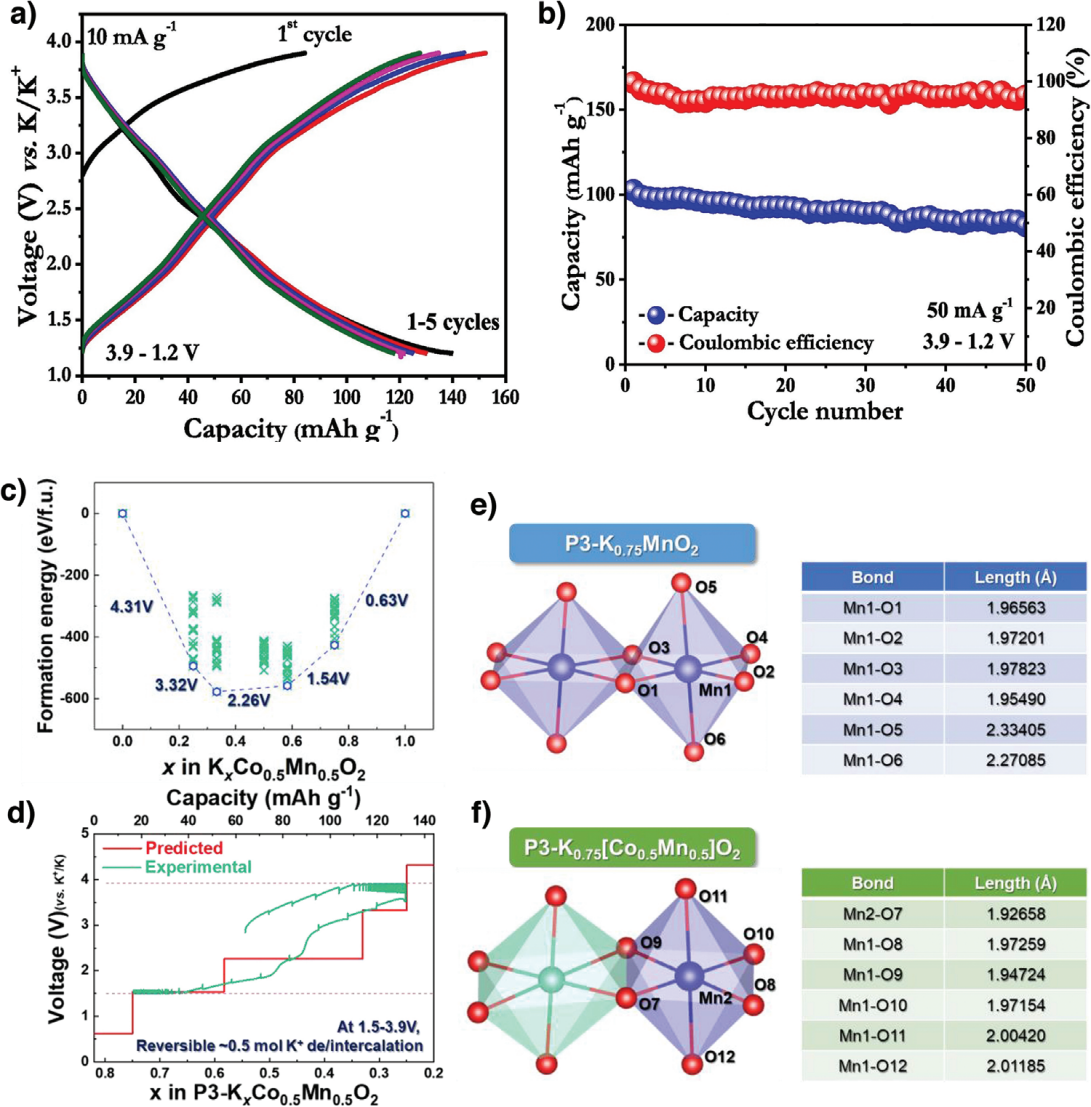
a) Charge–discharge curves of P3‐K_0.45_Mn_0.5_Co_0.5_O_2,_ b) cyclic stability of P3‐K_0.45_Mn_0.5_Co_0.5_O_2_. Reproduced with permission.^[^ ^81^ ^]^ Copyright 2019, Elsevier. c) Formation energy of P3‐K*_x_* [Co_0.5_Mn_0.5_]O_2_ (0 ≤ *x* ≤ 1), d) comparison of experimentally measured GITT charge/discharge curve and predicted voltage profile, and comparison of Mn—O bonding distances between e) P3‐K_0.75_MnO_2_ and f) P3‐K_0.75_[Co_0.5_Mn_0.5_]O_2_. Reproduced with permission.^[^ ^82^ ^]^ Copyright 2019, Elsevier.

Single transition‐metal oxides such as P3‐K_0.5_MnO_2_ and P2‐K_0.6_CoO_2_ exhibit multistep voltage profiles owing to multiple phase transitions and K^+^/vacancy ordering.^[^ ^47^, ^56^ ^]^ In the binary metal oxide compound P3‐K_0.45_Mn_0.5_Co_0.5_O_2_, the Mn and Co in the lattice structure suppress the K^+^/vacancy ordering and stabilize the cathode structure, resulting in a smooth voltage profile (**Figure** **9**a). The P3‐K_0.45_Mn_0.5_Co_0.5_O_2_ cathode delivered a specific capacity of 140 mAh g^−1^ involving both the Mn^3+^/Mn^4+^ and Co^3+^/Co^4+^ redox couples and exhibited cycling stability with 80% capacity retention after 50 cycles (Figure 9b).^[^ ^81^ ^]^ Mn^3+^‐induced J‐T distortion unidirectionally increases the MnO_6_‐octahedral Mn–O distance in discharged Mn‐based cathode materials. Choi et al. obtained P3‐K_0.54_[Co_0.5_Mn_0.5_]O_2_ in which Co^3+^ replaced half the Mn^3+^ and increased the Mn oxidation state above 3.5+, thus minimizing the J‐T distortions.^[^ ^82^ ^]^ First‐principles calculations predicted the formation energies of several stable intermediate phases as functions of K^+^‐ion content (Figure 9c).

The predicted voltage profile matched well with the experimental galvanostatic intermittent titration technique (GITT) measurements and exhibited a smooth voltage profile compared to nondoped K*_x_* MnO_2_ cathodes (Figure 9d). In addition, two types of Mn—O bonds were longer than the others in MnO_6_ octahedra in the pristine P3‐K_0.75_MnO_2_ structure. All the MnO_6_ octahedra Mn—O bond lengths are similar in the Co‐substituted P3‐K_0.75_[Co_0.5_Mn_0.5_]O_2_ structure (Figure 9e,f), which implies that Co substitution effectively mitigated the Mn^3+^‐induced J‐T structural distortion.^[^ ^82^ ^]^ The P3‐K_0.48_Mn_0.4_Co_0.6_O_2_ cathode exhibited a discharge capacity of 64 mAh g^−1^ and an average output of 3.0 V owing to the solid‐solution K^+^ deintercalation/intercalation mechanism. Furthermore, the cathode displayed stable cycling and good capacity retention (81%) after 180 cycles. These studies indicate that further optimizing the layered oxide Mn/Co content could lead to the design of high‐performance KIB cathodes.^[^ ^83^ ^]^

Briefly, the optimal amount of Co^3+^ substitution was found to effectively suppress the J–T distortions caused by Mn^3+^ and thus make possible to obtain smoother voltage curves without multiple steps. However, the Co is toxic and expensive which hampers its use in practical batteries.

#### 3.2.3 Mn/Ni‐Based Electrodes

**Figure 10:**
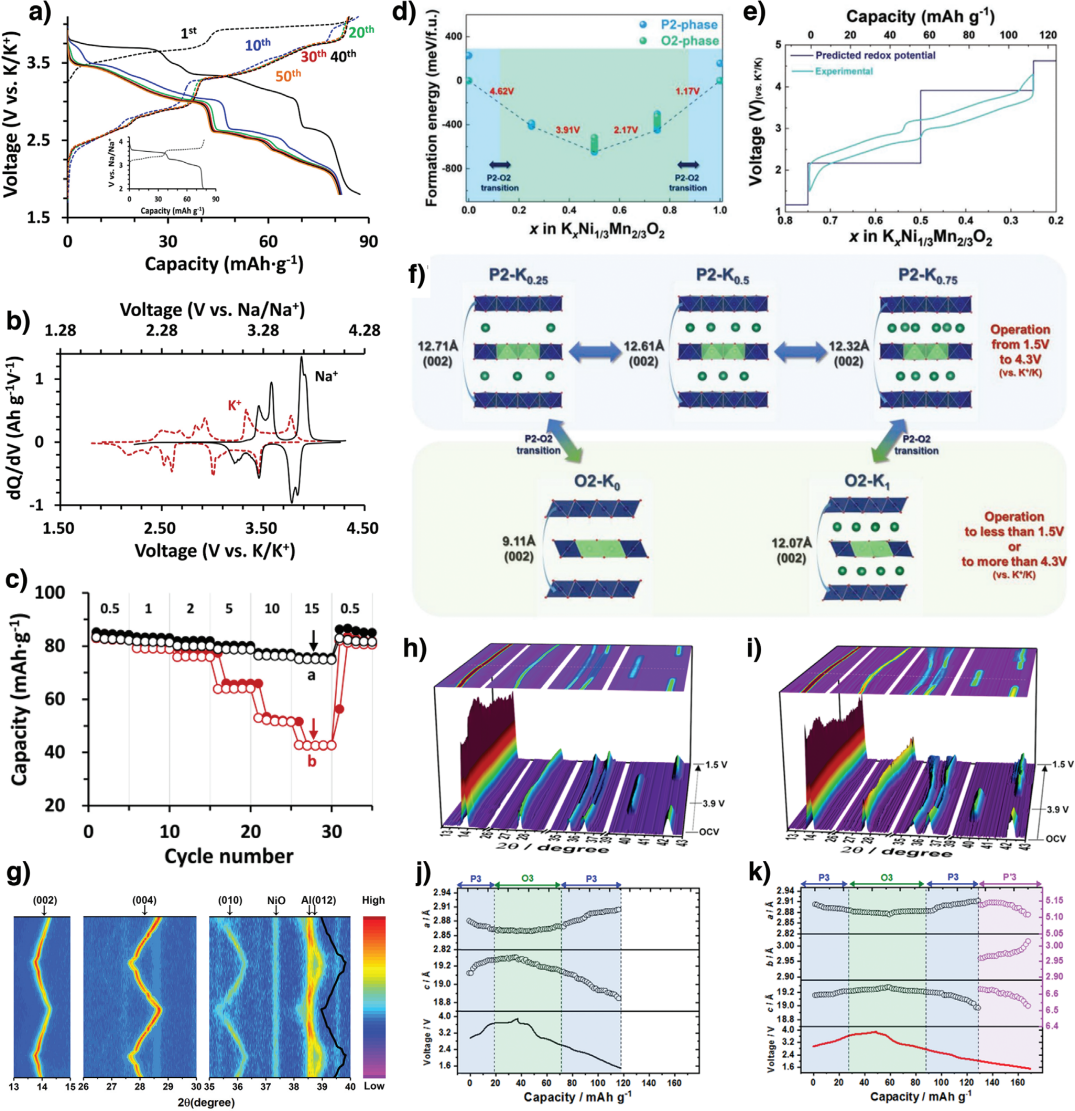
a) Charge–discharge profiles of NNMO during K^+^ exchange process, b) comparison of d*Q*/d*V* curves of NNMO in Na^+^ and K^+^ electrolytes. c) Comparison of rate capability of KNMO. Reproduced with permission.^[^ ^41^ ^]^ Copyright 2019, Elsevier. d) Formation energies of P2/O2‐K_x_[Ni_1/3_Mn_2/3_]O_2_ with various K contents. e) Comparison between experimentally measured charge/discharge curves and redox potentials predicted from first‐principles calculations, f) predicted structural change of P2/O2‐K*_x_* [Ni_1/3_Mn_2/3_]O_2_ as a function of K content. Reproduced with permission.^[^ ^84^ ^]^ Copyright 2020, Wiley. g) In situ XRD patterns of P2‐K_0.44_Ni_0.22_Mn_0.78_O_2_ electrode collected during the first and second charge/discharge at 10 mA g^−1^ in the voltage range of 1.5–4.0 V. Reproduced with permission.^[^ ^89^ ^]^ Copyright 2019, Wiley. Operando synchrotron XRD patterns and calculated lattice parameters for h,i) K_0.5_MnO_2_ and j,k) K_0.5_[Ni_0.1_Mn_0.9_]O_2_. Reproduced with permission.^[^ ^76^ ^]^ Copyright 2019, American Chemical Society.

Incorporating Ni^2+^ into K*_x_* MnO_2_ could replace the J‐T distortion‐active Mn^3+^ ions, and Ni^2+/4+^ redox couple in the high‐voltage region to contribute to high energy density. Nathan et al. developed a layered P2‐K_≈2/3_[Ni_1/3_Mn_2/3_]O_2_ (KNMO) cathode through electrochemical ion exchange from sodium compounds.^[^ ^41^ ^]^ When P2‐Na_0.64_[Ni_1/3_Mn_2/3_]O_2_ (NNMO) was continuously cycled in a K^+^‐containing electrolyte, nearly all the Na^+^ ions were progressively replaced by K^+^ ones. The KNMO exhibited more voltage steps than NNMO, indicating that frequent K^+^/vacancy ordering is required to transport larger K^+^ ions compared to Na^+^ ones (**Figure** **10**a,b). The higher K^+^‐ion preference at prismatic sites transitioned the P2 to O2 at a higher voltage (4.65 V vs K/K^+^). The decelerated P2–O2 transition and rapid diffusion during K^+^‐ion extraction enabled KNMO charging, even at a high (15 C) rate (2850 mA g^−1^). The cathode delivered a discharge capacity of 82 mAh g^−1^ and exhibited excellent rate capability by maintaining the P2 phase in a wide potential window 1.5–4.5 V versus K/K^+^ (Figure 10c).^[^ ^41^ ^]^ Adopting the same electrochemical ion‐exchange method, P2‐K_0.75_[Ni_1/3_Mn_2/3_]O_2_ was synthesized when ions were exchanged in a slightly wider range 1.5–4.3 V versus K/K^+^.^[^ ^84^ ^]^ The cathode delivered a high reversible capacity of 110 mAh g^−1^ at 20 mA g^−1^ and exhibited excellent cycling stability with 83% capacity retention over 500 cycles at 1400 mA g^−1^. The XANES measurements showed that P2‐K_0.75_[Ni_1/3_Mn_2/3_]O_2_ involves the Ni^2+^/Ni^4+^ redox couple, while Mn^4+^ is inactive during K^+^‐ion extraction/insertion. However, because the Mn^4+^ does provide structural stability, the material undergoes a single‐phase reaction that maintains the P2 phase in the operation voltage range. The experimental results were supported by first‐principles calculations. The calculated formation energies predicted that P2‐K*_x_* [Ni_1/3_Mn_2/3_]O_2_ was more stable than O2‐K*_x_* [Ni_1/3_Mn_2/3_]O_2_ (Figure 10d). As shown in Figure 10e, the predicted redox potential is in line with the experimentally measured charge–discharge profile. The predicted structural changes are plotted as functions of K^+^‐ion content in Figure 10f. When *x* approaches ≈ 0 or 1 in P2‐K*_x_* [Ni_1/3_Mn_2/3_]O_2_, the variation in the prismatic (P2) structure causes layer gliding along the *c*‐axis, resulting in the formation of octahedral (O2) structures.^[^ ^84^ ^]^ Similarly, Choi et al. prepared a high‐energy‐density Pʹ2‐K_0.83_[Ni_0.05_Mn_0.95_]O_2_ cathode by electrochemically ion‐exchanging Pʹ2‐Na_0.67_[Ni_0.05_Mn_0.95_]O_2_. The Pʹ2‐K_0.83_[Ni_0.05_Mn_0.95_]O_2_ cathode delivered a high specific capacity of 155 mAh g^−1^ and exhibited a high energy density of 420 Wh kg^−1^. Interestingly, the cathode demonstrated excellent structural stability by maintaining the Pʹ2 phase without transitioning to the OP4 one during K^+^‐ion deintercalation/intercalation compared with other Pʹ2‐based layered cathode materials for application to NIBs.^[^ ^85^, ^86^, ^87^ ^]^ Notably, the low activation barrier energy (271 meV) for K^+^‐ion transport enables the high‐rate performance of 78 mAh g^−1^ at 2600 mA g^−1^. Furthermore, the full cell assembled using the Pʹ2‐K_0.83_[Ni_0.05_Mn_0.95_]O_2_ cathode and a hard carbon anode delivered a capacity of 135 mAh g^−1^ and exhibited long‐term cycling stability with 80% capacity retention after 300 cycles.^[^ ^88^ ^]^

Zhang et al. used a solid‐state method to synthesize a P2‐K_0.44_Ni_0.22_Mn_0.78_O_2_ cathode, which delivered a specific capacity of 125.5 mAh g^−1^ at 10 mA g^−1^ and retained 67% of its initial capacity after 500 cycles. Notably, a trace of NiO impurities in the as‐synthesized material was electrochemically inactive during charging and discharging. The in situ XRD patterns (Figure 10g) show that this material undergoes a single‐phase transition and exhibits a small volumetric change (1.5%) upon K^+^‐ion extraction/insertion.^[^ ^89^ ^]^ Partially substituting Ni^2+^ for Mn^3+^ in P3‐K*_x_* MnO_2_ effectively mitigated the structural deterioration due to the Mn^3+^‐ion‐induced J‐T effect.^[^ ^76^ ^]^ The P3‐K_0.5_[Ni_0.1_Mn_0.9_]O_2_ cathode delivered a specific capacity of 121 mAh g^−1^ with 82% capacity retention after 100 cycles. The operando synchrotron XRD patterns revealed the structural evolution during K^+^‐ion extraction/insertion in the developed compounds (Figure 10h–k). P3‐K_0.5_[Ni_0.1_Mn_0.9_]O_2_ reversibly transitions among the phases P3, O3, P3, and Pʹ3 during charging and discharging. When the K^+^ ions were extracted, the interlayer distance increased owing to oxygen–oxygen repulsion, which is reflected in the *c*‐axis parameters (Figure 10k). The variation in the transition‐metal valence states (i.e., Ni^2+/4+^ and Mn^3+/4+^) influences the *a*‐axis parameter because of the bond between the transition metal and the oxygen in the layered compounds. Compared to pristine P3‐K_0.5_MnO_2_, Ni‐substituted P3‐K_0.5_[Ni_0.1_Mn_0.9_]O_2_ exhibited better structural stability because of its smaller lattice changes (Figure 10i,k).^[^ ^76^ ^]^

Bai et al. used a solid‐state method to synthesize a series of P3‐K_0.67_Mn_1−_ *_x_* Ni*_x_* O_2_ (*x* = 0, 0.08, 0.17, and 0.33) layered compounds. The P3‐K_0.67_Mn_0.83_Ni_0.17_O_2_ compound prepared using the optimal Ni‐content effectively suppressed the Mn^3+^‐induced J‐T distortions, reduced the structural deterioration, and eventually enhanced the cathode electrochemical performance. The P3‐K_0.67_Mn_0.83_Ni_0.17_O_2_ cathode delivered a specific capacity of 122 mAh g^−1^ at 20 mA g^−1^ and exhibited good cycling stability with 75% capacity retention at 500 mA g^−1^ after 200 cycles.^[^ ^77^ ^]^ K^+^/vacancy ordering limits K^+^‐ion diffusion kinetics and the practical capacity of layered compounds and causes layered oxides to exhibit step‐like voltage profiles and numerous CV‐curve redox peaks.^[^ ^41^ ^]^ Xiao et al. studied the effect of the K^+^‐ion content on the transformation from a K^+^/vacancy‐ordered structure to a K^+^/vacancy‐disordered one.^[^ ^90^ ^]^ The high K^+^‐ion content affects the interlayer K^+^–K^+^ electrostatic repulsion and reduces the K^+^‐ion site energy differences, thus breaking the K^+^/vacancy‐ordered structure. K^+^/vacancy‐disordered K_0.7_Mn_0.7_Ni_0.3_O_2_ exhibits much better rate performance and a higher discharge capacity than K^+^/vacancy‐ordered K_0.4_Mn_0.7_Ni_0.3_O_2_. The full cell assembled using the K_0.7_Mn_0.7_Ni_0.3_O_2_ cathode and a soft carbon anode delivered a capacity of 95.1 mAh g^−1^ at 100 mA g^−1^ and retained 86.4% of its initial capacity after 100 cycles.^[^ ^90^ ^]^

In summary, the partial substitution of Ni^2+^ replaced J–T active Mn^3+^ and suppressed the P2‐O2 phase transitions and structural degradation. Further, the Ni^2+/4+^ redox couple is able to provide high average voltage, which is beneficial for developing high‐energy density batteries. But the Mn/Ni‐based compounds commonly suffer from K^+^/vacancy ordering which leads to multiple voltage steps. The design of K^+^/vacancy disordered structures by tuning the elemental composition is found to be efficient strategy to improve the performance.

#### 3.2.4 Mn/Mg‐Based Electrodes

**Figure 11:**
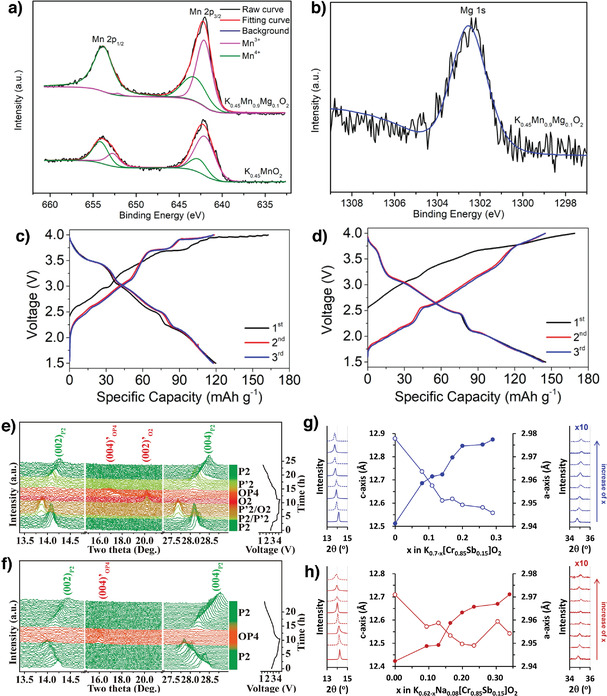
High‐resolution XPS spectra of a) Mn 2p and b) Mg 2p in H‐K_0.7_Mn_0.7_Mn_0.3_O_2_. Reproduced with permission.^[^ ^92^ ^]^ Copyright 2019, Wiley. Galvanostatic charge–discharge profiles of c) H‐K_0.7_MnO_2_ and d) H‐K_0.7_Mn_0.7_Mg_0.3_O_2_. Reproduced with permission.^[^ ^93^ ^]^ Copyright 2020, Elsevier. In situ XRD patterns and corresponding voltage–capacity curve of e) P2‐ K_5/9_MnO_2_ and f) P2‐ K_5/9_Mn_7/9_Ti_2/9_O_2_ at 10 mA g^−1^. Reproduced with permission.^[^ ^29^ ^]^ Copyright 2020, Elsevier. Dimensional changes at various states‐of‐charge in g) P2‐K_0.7_[Cr_0.85_Sb_0.15_]O_2_ and h) P2‐K_0.62_Na_0.08_[Cr_0.85_Sb_0.15_]O_2_. The solid and hollow circles denote changes in the *c*‐axis and *a*‐axis lengths, respectively. The XRD patterns for (002) and (010) peaks are shown in the left and right panels, respectively. The (010) peaks are magnified by 10. Reproduced with permission.^[^ ^12^ ^]^ Copyright 2020, Electrochemical Society.

Doping electrochemically inactive elements is an efficient strategy for improving the electrochemical performance of layered oxide cathodes. Although the nonredox behavior of dopants minimizes the overall material charge storage, dopants can stabilize crystal structures, suppress phase transitions, eliminate unwanted order–disorder transitions, and act as pillars to strengthen mechanical properties.^[^ ^91^ ^]^ Liu et al. partially substituted inactive Mg for Mn and stabilized the P3‐K_0.45_Mn_0.9_Mg_0.1_O_2_ structure during cycling. X‐ray photoelectron spectroscopy (XPS) indicated that substituting Mg^2+^ for Mn^3+^ could increase the Mn^4+^ content, which is beneficial for suppressing the J‐T distortion (**Figure** **11**a,b).^[^ ^92^ ^]^ The initial discharge capacities of the pristine K_0.45_MnO_2_ and doped K_0.45_Mn_0.9_Mg_0.1_O_2_ cathodes were 116.3 and 108 mAh g^−1^, respectively. K_0.45_Mn_0.9_Mg_0.1_O_2_ exhibited a lower initial capacity because electrochemically inert Mg^2+^ ions had partially substituted for some active Mn^3+^ ions. However, the doped K_0.45_Mn_0.9_Mg_0.1_O_2_ demonstrated improved cycling stability and rate performance compared to pristine K_0.45_MnO_2_ because substituting Mg^2+^ reduces the volumetric change, increases the lamellar spacing for rapid K^+^‐ion extraction/insertion, and contributes to the high‐rate performance.^[^ ^92^ ^]^ Similarly, Mg‐substituted hierarchical H‐K_0.7_Mn_0.7_Mg_0.3_O_2_ microparticles exhibited a high reversible capacity of 144.5 mAh g^−1^ at 20 mA g^−1^. The Mg‐substituted H‐K_0.7_Mn_0.7_Mg_0.3_O_2_ cathode displayed fewer voltage steps and less polarization than the pristine H‐K_0.7_MnO_2_ one (Figure 11c,d). Furthermore, the full cell assembled using the H‐K_0.7_Mn_0.7_Mg_0.3_O_2_ cathode and a hard carbon anode delivered a discharge capacity of 73.5 mAh g^−1^ at 100 mA g^−1^ and retained 75% of its initial capacity after 100 cycles.^[^ ^93^ ^]^

Xu et al. substituted inactive Ti^4+^ for active Mn^4+^ in P2‐K_5/9_MnO_2_ to prepare a series of P2‐K_5/9_Mn_1−_ *_x_* Ti*_x_* O_2_ (0 ≤ *x* ≤ 4/9) compounds.^[^ ^29^ ^]^ In situ XRD analysis revealed cathode structural changes during K^+^‐ion extraction and insertion. Pristine P2‐K_5/9_MnO_2_ displayed structural degradation and multiple transitions among the P2, Pʹ2, O2, OP4, Pʹ2, and P2 phases (Figure 11e). In contrast, the Ti‐doped P2‐K_5/9_Mn_7/9_Ti_2/9_O_2_ XRD pattern showed the opposite trend (Figure 11f), thus providing improved electrochemical performance. Therefore, Ti^4+^ effectively diminished TMO_2_ slab gliding and prevented the destructive P2‐O2 phase transition by promoting the highly reversible P2‐OP4 phase transition during charging and discharging.^[^ ^29^ ^]^ Nathan et al. synthesized inactive Sb^5+^‐doped P2‐K_0.70_[Cr_0.85_Sb_0.15_]O_2_ (KCSO) by a solid‐state method and then compared its electrochemical properties with those of P2‐K_0.62_Na_0.08_[Cr_0.85_Sb_0.15_]O_2_ prepared using electrochemical ion exchange.^[^ ^12^ ^]^ The ion‐exchange P2‐K_0.62_Na_0.08_[Cr_0.85_Sb_0.15_]O_2_ (IE‐KCSO) was obtained by cycling the P2‐Na_0.70_[Cr_0.85_Sb_0.15_]O_2_ sodium compound in the K^+^‐ion electrolyte. Notably, the IE‐KCSO contained Na^+^ ions, even after prolonged cycling, and demonstrated superior electrochemical performance by retaining 96% of its initial capacity after 100 cycles in contrast to only 76% capacity retention for KCSO. As shown in Figure 11g,h, a trace of Na^+^ ions remaining in IE‐KCSO causes smaller dimensional changes in IE‐KCSO and facilitates K^+^‐ion diffusion during charging and discharging, which contributes to the superior cyclability and rate capability.^[^ ^12^ ^]^ This result was further supported by a systematic study of the influence of Na^+^ substitution on the cathode electrochemical performance by Liu et al., who varied the Na content to synthesize layered K_0.67−_ *_x_* Na*_x_* Ni_0.17_Co_0.17_Mn_0.66_O_2_ (*x* ≤ 0.5) by coprecipitation followed by a solid‐state reaction.^[^ ^94^ ^]^ Reportedly, introducing Na^+^‐ions affects the crystal structure, the electrode morphology, and eventually the electrochemical behavior. Compared with pristine P3‐K_0.67_Ni_0.17_Co_0.17_Mn_0.66_O_2_, the Na^+^‐substituted compounds exhibited improved electrochemical performance. However, excess Na^+^‐ion substitution (*x* > 0.3) suppressed K^+^‐ion migration during charging and discharging; thus, an efficient cathode was obtained when the Na^+^‐ion content was suitably adjusted within a reasonable range.^[^ ^94^ ^]^

In summary, the doping of electrochemically inactive elements (Mg^2+^, Ti^4+^) is a useful strategy to suppress the structural transition and improve the long‐term cyclability of the layered compounds. The Mg^2+^ mitigated J–T distortions and increased interlayer spacing for K^+^ diffusion leading to high rate capability. The Ti^4+^ substitution prevented layer gliding and thus improved structural stability during charge/discharge. The excess doping of inactive elements decreases the specific capacity, so the dopant concentration must be carefully tuned for designing efficient electrode materials.

### 3.3 Multimetal‐Based Electrodes

Although the binary metal oxide cathodes demonstrate enhanced electrochemical performance compared to the single metal oxide systems, they could not meet the requirements of practical KIBs. Therefore, the design of new materials with multiple metals could combine the synergistic effect of different metals and thus leading to improved performance. The commercialization of ternary compounds LiNi*_x_* Co*_y_* Mn*_z_* O_2_, where *x* + *y* + *z* = 1 (NMC) and LiNi_0.85_Co_0.1_Al_0.05_O_2_ (NCA) for application to LIBs^[^ ^95^ ^]^ and Na–NCM layered cathodes for application to NIBs^[^ ^96^ ^]^ paved the way for designing K^+^‐containing ternary metal layered cathodes for application to KIBs. However, K^+^‐containing ternary metal layered cathodes may not be directly applicable to KIBs because larger K^+^ ions cause such cathodes to exhibit more structural evolution and complex electrochemical behavior. From the knowledge gained by exploring LIB and NIB cathodes, Liu et al. developed a K_0.67_Ni_0.17_Co_0.17_Mn_0.66_O_2_ layered ternary material. The cathode delivered a reversible capacity of 76.5 mAh g^−1^ and an average output of 3.1 V. The charge‐storage mechanism was accompanied by Mn^3+^/Mn^4+^ and Ni^2+^/Ni^4+^ redox reactions, while Co doping supposedly stabilized the cathode structure.^[^ ^97^ ^]^

**Figure 12:**
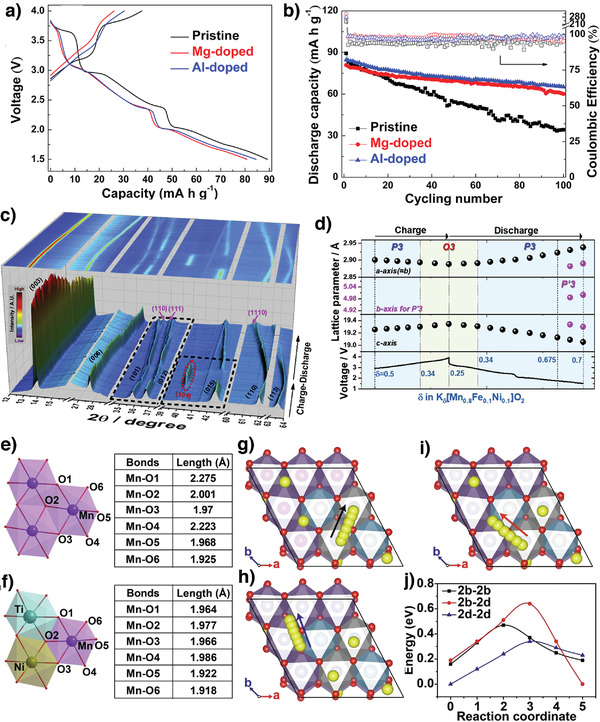
The electrochemical performances for the pristine P3‐K_0.45_Ni_0.1_Co_0.1_Mn_0.8_O_2_, Mg‐doped, and Al‐doped cathodes. a) Galvanostatic charge/discharge curves and b) cycling performance at 20 mA g^−1^. Reproduced with permission.^[^ ^99^ ^]^ Copyright 2020, Elsevier. c) Operando synchrotron XRD patterns for the P3‐K_0.5_[Mn_0.8_Fe_0.1_Ni_0.1_]O_2_ and d) the lattice parameters calculated from operando SXRD patterns during the charge–discharge processes. Reproduced with permission.^[^ ^101^ ^]^ Copyright 2020, Elsevier. e,f) Schematic from the DFT calculation for Mn–O bonding distances of K_0.6_MnO_2_ and K_0.6_Mn_0.8_Ni_0.1_Ti_0.1_O_2_, respectively. Predicted K^+^ diffusion paths and activation barrier energy in the K_0.6_Mn_0.8_Ni_0.1_Ti_0.1_O_2_ by first‐principles calculation, g) K_f_ to K_f_, h) K_f_ to K_e_, i) K_e_ to K_e,_ and j) corresponding energy barriers for the migration pathways displayed in (g–i) by using the NEB method. Reproduced with permission.^[^ ^103^ ^]^ Copyright 2021, Elsevier.

Furthermore, designing specially structured layered cathode materials can facilitate rapid K^+^‐ion transport owing to short diffusion paths and can accommodate stress induced by continuous K^+^‐ion extraction/insertion. The P3‐K_0.5_Mn_0.72_Ni_0.15_Co_0.13_O_2_ microspheres tested as cathodes exhibited improved electrochemical performance with an initial discharge capacity of 82.5 mAh g^−1^ and excellent cycling stability with 85% capacity retention after 100 cycles at 50 mA g^−1^.^[^ ^98^ ^]^ In another study, Dang et al. improved the P3‐K_0.45_Ni_0.1_Co_0.1_Mn_0.8_O_2_ electrochemical performance by doping Mg^2+^ and Al^3+^ into Mn sites.^[^ ^99^ ^]^ Mg^2+^/Al^3+^ doping increased the K^+^‐layer interlayer spacing, which may decrease K^+^ migration resistance during cycling. Compared to the pristine sample, the doped samples exhibited lower discharge capacities because of the lower Mn^3+/4+^ active redox content because Mg^2+^/Al^3+^ did not participate in charge compensation (**Figure** **12**a). However, Mg^2+^ and Al^3+^ doping improved the cathode cycling stability (Figure 12b) by minimizing the Mn^3+^‐induced J‐T distortion, enlarging the K^+^‐ion diffusion layer, and enhancing the cathode structural stability.^[^ ^99^ ^]^

Hwang et al. prepared P2‐K_0.75_[Mn_0.8_Ni_0.1_Fe_0.1_]O_2_ by electrochemical ion exchange, and the cathode demonstrated reversible K^+^‐ion (0.5 mol) storage and thus delivered a capacity of 110 mAh g^−1^ without transitioning through multiple phases in the range 1.5–3.9 V versus K/K^+^.^[^ ^100^ ^]^ The low K^+^‐ion diffusion activation barrier (580 meV) was predicted for P2‐K*_x_* [Mn_0.8_Ni_0.1_Fe_0.1_]O_2_ using nudged elastic band (NEB) calculations. Because P2‐K_0.75_[Mn_0.8_Ni_0.1_Fe_0.1_]O_2_ exhibited large 2D K^+^‐ion diffusion pathways and a low activation barrier, it also exhibited excellent rate capability. Moreover, the P2‐K_0.75_[Mn_0.8_Ni_0.1_Fe_0.1_]O_2_//hard carbon full cell exhibited excellent long‐term stability over 1000 continuous cycles.^[^ ^100^ ^]^ By varying the K^+^‐ion content, Choi et al. prepared P3‐K_0.5_[Mn_0.8_Fe_0.1_Ni_0.1_]O_2_ using a combustion‐assisted solid‐state reaction. Partially replacing Mn^3+^ with Fe^3+^ and Ni^2+^ increased the average Mn valence state to 3.75+, which mitigated the J‐T distortions and structural degradation. The structural evolution (Figure 12c,d) investigated using synchrotron XRD showed that the electrode undergoes reversible phase transitions such as P3‐O3 during charging and O3‐P3‐Pʹ3 during discharging. Because the reversibility slightly varied (≈4.1%) the structure, the P3‐K_0.5_[Mn_0.8_Fe_0.1_Ni_0.1_]O_2_ cathode delivered a high discharge capacity of 120 mAh g^−1^ and retained 74% of its initial capacity after 300 cycles.^[^ ^101^ ^]^

The P3 compounds usually transition from P3 to O3 during K^+^‐ion extraction, which slows K^+^‐ion mobility and rapidly degrades the cathode capacity and may be ascribed to a higher activation barrier within the O framework and a substantially contracted crystal structure. Interestingly, Fe^3+^/Ti^4+^‐codoped P3‐K_0.4_Fe_0.1_Mn_0.8_Ti_0.1_O_2_ demonstrated a solid–solution transition without an obvious P3‐O3 transition during K^+^‐ion extraction/insertion in voltage window 1.8–4.0 V versus K/K^+^. The P3‐K_0.4_Fe_0.1_Mn_0.8_Ti_0.1_O_2_ cathode delivered an initial capacity of 117 mAh g^−1^ and exhibited long stability over 300 cycles and negligible volumetric change (0.5%).^[^ ^102^ ^]^ Similarly, the P2‐K_0.6_Mn_0.8_Ni_0.1_Ti_0.1_O_2_ solid solution did not exhibit any other phase transition (including OP4 or O2) and even charged to a high voltage of 4.2 V. In contrast, pristine P2‐K_0.6_MnO_2_ exhibited complex P2‐OP4‐*X* phase transitions during K^+^‐ion extraction.^[^ ^103^ ^]^ Furthermore, complex phase transitions considerably changed the pristine cathode *c*‐axis lattice parameter (Δ*c* = 31%), whereas the doped P2‐K_0.6_Mn_0.8_Ni_0.1_Ti_0.1_O_2_ cathode exhibited much less variation in the *c*‐axis parameter (Δ*c* = 2.4%). Clearly, Ni^2+^/Ti^4+^ doping not only mitigates J‐T distortions (Figure 12e,f) but also maintains the layer structural integrity, thereby improving the high‐voltage performance. Three possible K^+^‐ion migration pathways predicted using the climbing‐image nudged elastic band (CI‐NEB) method are shown in Figure 12g–i.^[^ ^103^ ^]^

Recently, Liu et al. studied the influence of Mg^2+^/Ni^2+^ codoping on the K*_x_* MnO_2_ electrochemical performance and synthesized various K_1/2_Mn*_x_* Mg_(1−_ *_x_*_)/2_Ni_(1−_ *_x_*_)/2_O_2_ composite compounds (*x* = 1, 9/10, 5/6, and 2/3) using the sol–gel method. Mg^2+^/Ni^2+^ cosubstitution remarkably influenced the K*_x_* MnO_2_ crystal structure. At low concentrations, Mg^2+^ and Ni^2+^ ions reportedly prefer to occupy the TM layer and begin to enter the K^+^‐ion layer at higher concentrations.^[^ ^104^ ^]^ The K^+^‐ion layer Mg^2+^ or Ni^2+^ ions serve as a pillar to prevent layer gliding, and Mg–Ni pinning suppresses multiphase transition in K*_x_* MnO_2_ during K^+^‐ion extraction/insertion.^[^ ^105^ ^]^ Designing high‐voltage K^+^‐ion‐containing layered cathodes to achieve high‐energy‐density KIBs is currently a hot research topic. Masese et al. reported a unique honeycomb layered K_2_Ni_2_TeO_6_ cathode, which exhibited a high average output of 3.6 V versus K/K^+^. The Ni^2+^/Ni^4+^ redox potential increased because of the [TeO_6_]^6−^ moiety induction, which is more electronegative than O_2_^2−^.^[^ ^106^ ^]^ In addition, they modified the tellurium content and synthesized a P2‐K_2/3_Ni_1/3_Co_1/3_Te_1/3_O_2_ multitransitional metal oxide cathode, which although delivered a low discharge capacity, displayed a high output of 4.3 V versus K/K^+^ and is by far the highest voltage ever recorded for KIB layered cathodes.^[^ ^107^ ^]^ A summary of the synthesis method and electrochemical performance of layered oxide cathodes in half‐cell KIBs and full‐cell KIBs are listed in **Tables** **1** and **2**, respectively.

**Table 1:** Electrochemical performance of layered oxide cathodes in half‐cell KIBs

Material	Synthesis method	Electrolyte	Voltage window [V]	Discharge capacity [mAh g^−1^]/ current density [mAg^−1^]	Capacity retention [%]/current density [mA g^−1^]/cycle	Refs.
K_0.3_MnO_2_	Thermal decomposition	1.5 m KFSI in EC:DMC	1.5–3.5	70/27.9	57/27.9/685	[45]
P3‐K_0.45_MnO_2_	Coprecipitation	0.8 _M_ KPF_6_ in EC:DEC	1.5–4.0	128.6/20	70.8/20/100	[46]
P3‐K_0.5_MnO_2_	Solid state	0.7 _M_ KPF_6_ in EC:DEC	1.5–3.9	106/5	70/20/50	[47]
P3‐K_0.5_MnO_2_	Two‐step self‐templating	0.8 _M_ KPF_6_ in EC:DEC	1.5–3.9	104/10	89.1/200/400	[48]
P2‐K_0.67_MnO_2_	Sol–gel	6.0 _M_ KFSI/diglyme (G2)	1.7–4.0	78/50	90.5/50/300	[53]
K_1.39_Mn_3_O_6_	Solid state	0.8 _M_ KPF_6_ in EC:DEC	1.5–4.0	110/10	94.9/50/100	[54]
P2‐K_0.41_CoO_2_	Solid state	1.0 _M_ KFSI in EC:DEC	2.0–3.9	57/11.8	96/11.8/30	[43]
P3‐K_2/3_CoO_2_	Solid state	1.0 _M_ KFSI in EC:DEC	2.0–3.9	60/11.5	91/11.5/30	[43]
P2‐K_0.6_CoO_2_	Solid state	0.7 _M_ KPF_6_ in EC:DEC	1.7–4.0	80/2	60/100/120	[56]
P2‐K_0.6_CoO_2_	Two‐step self‐templating	0.9 _M_ KPF_6_ in EC:DEC	1.7–4.0	82/10	87/40/300	[57]
O3‐KCrO_2_	Solid state	0.7 _M_ KPF_6_ in EC:DEC	1.5–4.0	92/5	67/10/100	[40]
O3‐KCrS_2_	Solid state	1.0 _M_ KFSI in EC:DEC	1.8–3.0	71/8.65	90/173/1000	[61]
P3‐K_0.69_CrO_2_	Electrochemical ion exchange	0.5 _M_ KPF_6_ in EC:DEC	1.5–3.8	100/10	65/100/1000	[66]
P′3‐K_0.8_CrO_2_	Solid state	0.5 _M_ KPF_6_ in EC:DEC	1.5–3.9	91/10.9	99/218/300	[42]
K_0.5_V_2_O_5_	Hydrothermal	1.0 _M_ KFSI in EC:DEC	1.5–3.8	87.5/20	81/100/250	[71]
*δ*‐K_0.42_V_2_O_5_·0.25H_2_O	Two‐step synthesis	0.8 _M_ KPF_6_ in EC:DEC	2.0–4.3	226/20	74/20/50	[72]
K_2_V_3_O_8_	Hydrothermal	7.0 _M_ KFSI in EC:DEC	1.5–4.5	107.8/10	73/10/50	[73]
K_0.83_V_2_O_5_	Chemistry route	7.0 _M_ KFSI in EC:DEC	1.5–4.3	90/10	86/10/200	[74]
K_0.7_Fe_0.5_Mn_0.5_O_2_	Electrospinning	0.8 _M_ KPF_6_ in EC:DEC	1.5–4.0	178/20	85/500/200	[78]
P2‐K_0.65_Fe_0.5_Mn_0.5_O_2_	Solvent thermal	0.9 _M_ KPF_6_ in EC:DEC	1.5–4.2	151/20	78/100/350	[44]
P3‐K_0.45_Mn_0.8_Fe_0.2_O_2_	Solid state	0.8 _M_ KPF_6_ in EC:DEC	1.5–4.0	106.2/20	77/20/100	[79]
K_0.4_Fe_0.5_Mn_0.5_O_2_	Solid state	1.0 _M_ KFSA in Pyr13FSA	1.3–3.8	120/12.5	85/12.5/50	[80]
P3‐K_0.45_Mn_0.5_Co_0.5_O_2_	Sol–gel	0.8 _M_ KPF_6_ in EC:DEC	1.2–3.9	140/10	80/50/50	[81]
P3‐K_0.54_[Co_0.5_Mn_0.5_]O_2_	Combustion	0.5 _M_ KPF_6_ in EC:DEC	1.5–3.9	120.4/20	85/20/100	[82]
P3‐K_0.48_Mn_0.4_Co_0.6_O_2_	Solid‐state	0.5 _M_ KPF_6_ in EC:DEC	1.0–4.2	64/6	81/12/180	[83]
P2‐K_≈2/3_[Ni_1/3_Mn_2/3_]O_2_	Electrochemical ion exchange	0.5 _M_ KPF_6_ in EC:DEC	1.5–4.5	82/86	99/86/100	[41]
P2‐K_0.75_[Ni_1/3_Mn_2/3_]O_2_	Electrochemical ion exchange	0.5 _M_ KPF_6_ in EC:DEC	1.5–4.3	110/20	86/20/300	[84]
P′2‐K_0.83_[Ni_0.05_Mn_0.95_]O_2_	Electrochemical ion exchange	0.5 _M_ KPF_6_ in EC:DEC	1.5–4.3	155/52	77/520/500	[88]
P2‐K_0.44_Ni_0.22_Mn_0.78_O_2_	Solid state	0.8 _M_ KPF_6_ in EC:DEC	1.5–4.0	125.5/10	67/200/500	[89]
P3‐K_0.5_[Ni_0.1_Mn_0.9_]O_2_	Combustion	0.5 _M_ KPF_6_ in EC:DEC	1.5–3.9	121/10	82/10/100	[76]
P3‐K_0.67_Mn_0.83_Ni_0.17_O_2_	Solid state	0.8 _M_ KPF_6_ in EC:DEC	1.5–3.8	122/20	75/500/200	[77]
P3‐K_0.7_Mn_0.7_Ni_0.3_O_2_	Solid state	0.8 _M_ KPF_6_ in EC:DEC	2.0–3.9	125.4/100	93.6/100/150	[90]
P3‐K_0.45_Mn_0.9_Mg_0.1_O_2_	Solid state	0.8 _M_ KPF_6_ in EC:DEC	1.5–4.0	108/20	74.8/20/100	[92]
K_0.7_Mn_0.7_Mg_0.3_O_2_	Resorcinol–formaldehyde	0.8 _M_ KPF_6_ in EC:DEC	1.5–4.0	144.5/20	82.5/100/400	[93]
P2‐K_0.7_[Cr_0.85_Sb_0.15_]O_2_	Solid state	0.5 _M_ KPF_6_ in EC:DEC	1.5–4.3	70/15.4	76/77/100	[12]
P2‐K_0.62_Na_0.08_[Cr_0.85_Sb_0.15_]O_2_	Electrochemical ion exchange	0.5 _M_ KPF_6_ in EC:DEC	1.5–4.3	78/15.4	96/77/100	[12]
P3/P2‐ K_0.37_Na_0.3_Ni_0.17_Co_0.17_Mn_0.66_O_2_	Coprecipitation	0.8 _M_ KPF_6_ in EC:DEC	2.0–4.3	86.1/20	62/100/100	[94]
P3‐K_0.67_Ni_0.17_Co_0.17_Mn_0.66_O_2_	Coprecipitation	0.8 _M_ KPF_6_ in EC:DEC	2.0–4.3	76.5/20	87/20/100	[97]
P3‐K_0.5_Mn_0.72_Ni_0.15_Co_0.13_O_2_	Solvothermal	0.8 _M_ KPF_6_ in EC:DEC	1.5–4.0	82.5/10	85/50/100	[98]
P3‐K_0.45_Ni_0.1_Co_0.1_Al_0.05_Mn_0.75_O_2_	Solid state	0.5 _M_ KPF_6_ in EC:DEC	1.5–4.0	84.5/20	77.4/20/100	[99]
P2‐K_0.75_[Mn_0.8_Ni_0.1_Fe_0.1_]O_2_	Electrochemical ion exchange	0.5 _M_ KPF_6_ in EC:DEC	1.5–3.9	110/10	70/100/200	[100]
P3‐K_0.5_[Mn_0.8_Fe_0.1_Ni_0.1_]O_2_	Combustion	0.5 _M_ KPF_6_ in EC:DEC	1.5–3.9	120/50	74/50/300	[101]
P3‐K_0.4_Fe_0.1_Mn_0.8_Ti_0.1_O_2_	Solid state	0.5 _M_ KPF_6_ in EC:DEC	1.8–4.0	117/20	74/200/300	[102]
P2‐K_0.6_Mn_0.8_Ni_0.1_Ti_0.1_O_2_	Solid state	0.5 _M_ KPF_6_ in EC:DEC	1.5–4.2	118/10	88/200/100	[103]
P3‐K_1/2_Mn_5/6_Mg_1/12_Ni_1/12_O_2_	Solid state	0.5 _M_ KPF_6_ in EC:DEC	1.5–3.9	83.3/120	70.4/120/200	[105]
P2‐Na_0.84_CoO_2_	Solution combustion	0.8 _M_ KPF_6_ in EC:DEC	2.0–4.0	82/10	58/20/50	[108]
O3‐Na_0.9_Cr_0.9_Ru_0.1_O_2_	Solid state	0.8 _M_ KPF_6_ in EC:DEC	1.5–3.8	100.6/10	81.2/500/500	[110]
P′3‐Na_0.52_CrO_2_	Solid state	1.0 _M_ KFSI in EC:DEC	2.0–3.6	88/12.5	67/500/200	[111]
P3‐K_0.48_Ni_0.2_Co_0.2_Mn_0.6_O_2_	Solid state	0.8 _M_ KPF_6_ in EC:PC	1.5–4.2	57/40	76.2/40/150	[112]

**Table 2:** Electrochemical properties of layered oxide cathodes in full‐cell KIBs

Material	Anode	Electrolyte	Voltage window [V]	Discharge capacity [mAh g^−1^]/current density [mA g^−1^]	Capacity retention [%]/current density [mA g^−1^]/cycle	Refs.
K_0.3_MnO_2_	Hard carbon & Carbon black	1.5 _M_ KFSI in EC:DMC	0.5–3.4	90/32	50/32/100	[45]
P3‐K_0.5_MnO_2_	Graphite	0.8 _M_ KPF_6_ in EC:DEC	0.5–3.4	60.2/10	66.7/10/50	[48]
P2‐K_0.6_CoO_2_	Graphite	0.7 _M_ KPF_6_ in EC:DEC	1.5–4.0	52/3	52/3/5	[56]
P2‐K_0.6_CoO_2_	Hard carbon	0.9 _M_ KPF_6_ in EC:DEC	0.5–3.8	71/30	80/30/100	[57]
K_0.83_V_2_O_5_	Graphite	7.0 _M_ KFSI in EC:DEC	0.5–3.9	75/10	80/10/30	[74]
O3‐KCrO_2_	Graphite	0.7 _M_ KPF_6_ in EC:DEC	0.5–4.0	97/5	53/5/10	[40]
K_0.7_Fe_0.5_Mn_0.5_O_2_	Soft carbon	0.8 _M_ KPF_6_ in EC:DEC	0.5–3.5	82/40	76/100/250	[78]
P3‐K_0.45_Mn_0.8_Fe_0.2_O_2_	Super P	0.8 _M_ KPF_6_ in EC:DEC	0.5–3.5	55/50	76/50/50	[79]
P2‐K_0.65_Fe_0.5_Mn_0.5_O_2_	Hard carbon	0.9 _M_ KPF_6_ in EC:DEC	0.5–3.5	75/100	80/100/100	[44]
P3‐K_0.54_[Co_0.5_Mn_0.5_]O_2_	Hard carbon	0.5 _M_ KPF_6_ in EC:DEC	0.5–3.6	96/20	82/20/100	[82]
P′2‐K_0.83_[Ni_0.05_Mn_0.95_]O_2_	Hard carbon	0.5 _M_ KPF_6_ in EC:DEC	0.5–4.0	135/52	80/52/300	[88]
P2‐K_0.44_Ni_0.22_Mn_0.78_O_2_	Soft carbon	0.8 _M_ KPF_6_ in EC:DEC	0.5–3.5	70/50	90/50/500	[89]
P3‐K_0.7_Mn_0.7_Ni_0.3_O_2_	Soft carbon	0.8 _M_ KPF_6_ in EC:DEC	1.1–3.5	95/100	86.4/100/100	[90]
K_0.7_Mn_0.7_Mg_0.3_O_2_	Hard carbon	0.8 _M_ KPF_6_ in EC:DEC	0.5–3.5	73.5/100	75/100/100	[93]
P2‐K_0.75_[Mn_0.8_Ni_0.1_Fe_0.1_]O_2_	Hard carbon	0.5 _M_ KPF_6_ in EC:DEC	0.5–3.5	60/20	60/20/1000	[100]
P3‐K_0.5_[Mn_0.8_Fe_0.1_Ni_0.1_]O_2_	Hard carbon	0.5 _M_ KPF_6_ in EC:DEC	0.5–3.6	113/50	89/50/150	[101]
P3‐K_0.4_Fe_0.1_Mn_0.8_Ti_0.1_O_2_	Soft carbon	0.5 _M_ KPF_6_ in EC:DEC	0.5–3.8	107/50	62/50/300	[102]

### 3.4 Na‐Based Cathodes for K^+^‐Ion Storage

**Figure 13:**
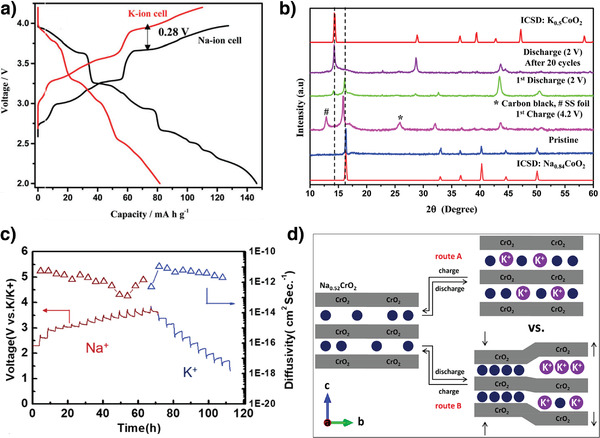
a) Charge–discharge profiles of P2‐N_0.84_CoO_2_ in Na‐ion cell and K‐ion cell. b) Ex situ XRD measurements of the pristine, charged, and discharged electrodes depicting the successful desodiation and potassiation of the pristine compound to form K_0.5_CoO_2_. Reproduced with permission.^[^ ^108^ ^]^ Copyright 2017, Royal Society of Chemistry. c) The diffusivity is calculated from GITT analysis for Na_0.9_Cr_0.9_Ru_0.1_O_2_. Reproduced with permission.^[^ ^110^ ^]^ Copyright 2019, Royal Society of Chemistry. d) Schematic diagram depicting two possible routes for reversible K^+^‐insertion/deinsertion in P′3‐Na_0.52_CrO_2_. Small spheres (blue) represent Na^+^ ions. Arrows indicate the direction of contraction/expansion along the *c*‐axis with K^+^‐insertion. Reproduced with permission.^[^ ^111^ ^]^ Copyright 2018, American Chemical Society.

In addition to K^+^‐ion‐containing layered cathodes, there have been a few reports on Na^+^‐containing layered materials for efficient K^+^‐ion storage. For example, Sada et al. demonstrated reversible K^+^‐ion intercalation in P2‐Na_0.84_CoO_2_ wherein the cathode delivered a reversible capacity of 82 mAh g^−1^ (**Figure** **13**a). The P2‐Na_0.84_CoO_2_ cathode was initially charged in a K^+^‐ion‐containing electrolyte for desodiation. During the subsequent discharge, K^+^ ions started replacing Na^+^ ones through progressive intercalation, and Na_0.34_K_0.5_CoO_2_ was formed after several charge–discharge cycles (Figure 13b).^[^ ^108^ ^]^ Furthermore, they developed another layered material (Na_2_Mn_3_O_7_) as a K^+^‐ion‐storage host structure. The Na_2_Mn_3_O_7_ cathode delivered a high capacity of 152 mAh g^−1^ and an energy density of 320 Wh kg^−1^.^[^ ^109^ ^]^

In another study, O3‐Na_0.9_Cr_0.9_Ru_0.1_O_2_ exhibited enhanced K^+^‐ion diffusion comparable and even superior to Na^+^‐ion diffusion (Figure 13c).^[^ ^110^ ^]^ During Na^+^ extraction, Na^+^ ions do not directly diffuse in O3 crystals, and Na^+^ ions diffusing through tetrahedral interstitial sites must overcome the high energy barrier therein. During K^+^‐ion intercalation, on the other hand, K^+^ ions diffuse from prismatic sites to others in a large interlayer space. The Ru‐substitution‐induced structural support and rapid K^+^‐ion diffusion kinetics enabled the Na_0.9_Cr_0.9_Ru_0.1_O_2_ to exhibit an excellent capacity retention of 81.2% after 500 cycles at a high rate (5 C).^[^ ^110^ ^]^ Naveen et al. developed Pʹ3‐Na_0.52_CrO_2_ as a KIB cathode, which delivered a specific capacity of 88 mAh g^−1^ and an average discharge potential of 2.95 V versus K/K^+^.^[^ ^111^ ^]^ In contrast to Na_0.84_CoO_2_,^[^ ^108^ ^]^ the Pʹ3‐Na_0.52_CrO_2_ exhibited uniquely distributed Na^+^ and K^+^ ions, forming a biphasic structure during K^+^‐ion insertion (Figure 13d). The reversible transition between monophasic (Na_0.5_CrO_2_) and biphasic NaCrO_2_/K_0.6_Na_0.17_CrO_2_ during cathode charging and discharging, respectively, decreased the volumetric changes and shortened the K^+^‐ion diffusion path, which contributed to the high‐rate and cycling performance.^[^ ^111^ ^]^ These studies provide insights for applying current Na^+^‐ion‐containing layered materials to K^+^‐ion storage. Further investigations on Na^+^/K^+^‐ion‐containing cathode materials are required to develop efficient KIBs.

### 3.5 Hybrid Potassium‐Ion Capacitor (KIC)

**Figure 14:**
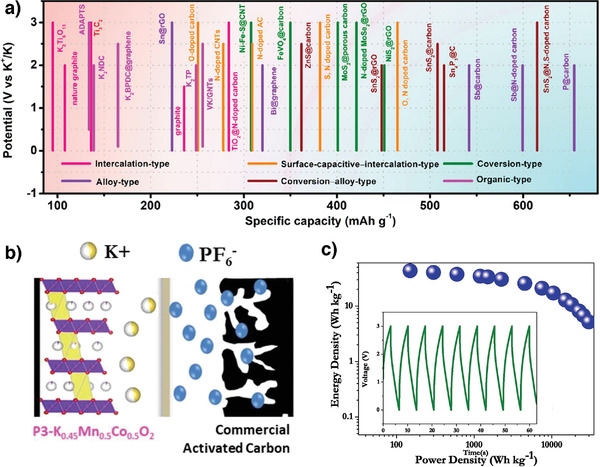
a) Various battery‐type anode materials tested in KICs. Reproduced with permission.^[^ ^122^ ^]^ Copyright 2021, American Chemical Society. b) Schematic illustration of a KIC fabricated with a battery‐type K_0.45_Mn_0.5_Co_0.5_O_2_ cathode and AC anode. c) Ragone plot of K_0.45_Mn_0.5_Co_0.5_O_2_//AC device. Inset: First few charge/discharge cycles at 10 A g^−1^. Reproduced with permission.^[^ ^81^ ^]^ Copyright 2019, Elsevier.

Supercapacitors are a class of energy storage systems that exhibit high power density and superior cycle life to secondary batteries but have comparatively low energy density.^[^ ^113^, ^114^, ^115^ ^]^ In recent years, metal‐ion hybrid capacitors (MICs) have been developed that bridge the gap between batteries and supercapacitors by combining battery‐type anodes with capacitor‐type cathodes. Numerous different MICs have been reported in the literature including the lithium‐ion (LIC), sodium‐ion (NIC), potassium‐ion (KIC) and zinc‐ion capacitors (ZIC), among others.^[^ ^116^, ^117^, ^118^, ^119^ ^]^ Comte et al. reported the first hybrid KIC using graphite (battery type) as the anode, activated carbon (capacitor type) as the cathode and a 0.8 m KPF_6_ EC:DMC electrolyte.^[^ ^120^ ^]^ During the charging process, the K^+^ ions are intercalated into the anode while the PF_6_^−^ anions in the electrolyte are adsorbed on the cathode surface, forming an electric double layer. During the discharge process, the K^+^ ions are deintercalated from anode and the PF_6_^−^ anions are released from cathode surface diffuse back to the electrolyte.^[^ ^121^ ^]^ Most current research efforts are focused on developing battery‐type anode materials for KICs. Such materials include carbon‐based materials, metal oxides, metal chalcogenides, MXenes and organic materials that follow different storage mechanisms such as intercalation, conversion, and alloying (**Figure** **14**a).^[^ ^122^, ^123^ ^]^ Conversely, carbon‐based capacitor‐type cathodes are extensively studied, but literature reports on the battery‐type cathodes for KIC are rare, perhaps because potassium‐ion storage systems are in an early development stage and suitable battery‐type cathodes have not yet been explored.^[^ ^124^ ^]^ Although the anode materials exhibit superior capacities, they must be combined with suitable cathode materials to achieve high energy and power density devices. Ramasamy et al.^[^ ^81^ ^]^ reported a non‐aqueous KIC consisting of a layered P3‐K_0.45_Mn_0.5_Co_0.5_O_2_ battery‐type cathode combined with a commercial activated carbon (AC) anode (Figure 14b). The constructed KIC delivered a high energy and power density of 43 Wh kg^−1^ and 30 kW kg^−1^, respectively, and retained 88% of its energy density over 30 000 cycles at 10 A g−1, demonstrating excellent cyclic stability (Figure 14c). The mass ratio between anode and cathode in the full cell KIC must be balanced according to their working potentials to obtain high energy/power density. Wei et al.^[^ ^125^ ^]^ fabricated an aqueous KIC with a layered K_0.296_Mn_0.926_O_2_ cathode and AC anode. The K_0.296_Mn_0.926_O_2_//AC displayed high energy and power densities of 70 W kg^−1^ and 6000 W kg^−1^, respectively, and a capacitance retention of 89.3% after 10 000 cycles. The unique layered structure facilities the rapid diffusion of K^+^ ions and provides space for charge storage.

The aforementioned studies demonstrate promising methods to construct aqueous or non‐aqueous KICs with battery‐type cathodes and also suggest numerous opportunities for the design of novel electrodes for high‐performance KICs. The rates of cation insertion/desertion and anion adsorption/desorption control the energy and power densities of KICs. The development of KICs is hampered by the sluggish kinetics of the anode and cathode materials; therefore, designing nanostructure and morphology‐controlled electrodes, developing a suitable electrolyte, and an in‐depth understanding of the reaction kinetics and energy storage mechanisms are essential factors for the realization of practical high‐performance KICs.

## 4 Synthesis of Layered Metal Oxide Electrodes

The methods used in the synthesis of active materials for various energy storage devices greatly influence their properties. The reaction parameters, including the temperature, time, pH, and precursors, affect the structural properties of the material, including the crystallinity, particle size, surface morphology, composition, and phase purity.^[^ ^126^, ^127^, ^128^ ^]^ In addition, the electrochemical properties of the electrode materials, such as the initial capacity, rate capability, and cyclic stability, are also influenced by the preparative techniques.^[^ ^129^, ^130^, ^131^ ^]^ Electrode materials have been synthesized by various techniques, including solid‐sate coprecipitation, sol–gel, hydrothermal/solvothermal, combustion, and electrochemical‐ion exchange reactions, among others. Further, fine‐tuning the reaction parameters and employing multiple synthetic routes may yield electrode materials with the desired stoichiometry, phase, morphology, and electrochemical properties.^[^ ^132^, ^133^, ^134^ ^]^ The remainder of Section 4 will discuss some of the synthetic routes used to produce KIB electrodes and their influence on the properties of the electrodes.

### 4.1 Solid‐State Reaction

The solid‐state reaction route, which is most commonly used to prepare layered metal‐oxide cathodes for KIBs (Table 1), is a simple method that involves the physical mixing (sometimes solution mixing) and sintering of potassium and transition metal precursors (oxides or acetates) to obtain the final products. For instance, Kim et al.^[^ ^47^ ^]^ synthesized P3‐K_0.5_MnO_2_ using K_2_CO_3_ and Mn_2_O_3_ precursors via a conventional solid‐state route. The stoichiometrically mixed powders were ball milled for 4 h before the pelletized mixture was calcined at 800 ° C for 12 h. The required phase structure and crystallinity can be obtained by selecting the appropriate precursors and controlling the calcination temperature. Hironaka et al.^[^ ^43^ ^]^ synthesized P3‐K_2/3_CoO_2_ and P2‐K_0.41_CoO_2_ phases by varying the precursors, composition, and reaction temperatures. P3‐K_2/3_CoO_2_ was synthesized by heating a mixture of KOH and Co(OH)_2_ at 400 ° C for 24 h, while P2‐K_0.41_CoO_2_ was obtained by firing a mixture of KOH and Co_3_O_4_ at 600 ° C for 4 h. The first charge profiles of the P3 and P2 compounds differed, but otherwise, the two compounds showed similar voltage changes, indicating that the potential primarily depends on the K^+^/vacancy ordering rather than the crystal structure. Kim et al.^[^ ^40^ ^]^ synthesized stoichiometric O3‐KCrO_2_ by sintering a pelletized mixture of KN_3_, KNO_3_, and Cr_2_O_3_ under controlled atmosphere and temperature conditions for 42 h. The numerous phase changes in the stoichiometric layered potassium cathode prevented the entire K^+^ content from being utilized during the charge/discharge process, limiting the cyclability and rate capability of the material. Element doping is one of the most efficient strategies to mitigate the lattice changes and thus improve the structural stability and durability of the cathodes. Liu et al. synthesized a series of P3‐K_0.45_Mn_1‐_ *_x_* Fe*_x_* O_2_ (*x* ≤ 0.5) to investigate the effects of Fe doping.^[^ ^79^ ^]^ A stoichiometric mixture of K_2_CO_3_, Mn_2_O_3_, and Fe_2_O_3_ was ball milled in a small amount of ethanol for 12 h. Notably, an excess of K_2_CO_3_ (5 wt%) was added to compensate for the loss of potassium at high temperature. The dried mixtures were pelletized and sintered at 850 ° C for 15 h. A cathode with superior performance was obtained with a doping level of *x* = 0.2, whereas excessive doping (*x* > 0.4) resulted in the formation of some impurities in the cathode, indicating that optimizing the dopant concentration improved the structural and electrochemical properties of the cathode. Xu et al.^[^ ^103^ ^]^ reported the synthesis of P2‐K_0.6_Mn_0.8_Ni_0.1_Ti_0.1_O_2_ with multiple elements doping. A pelletized mixture of K_2_CO_3_ (3 mol% excess), Mn_2_O_3_, NiO_2_, and TiO_2_ was calcined at 1000 ° C for 15 h. The substitution of Ni^2+^ and Ti^4+^ effectively suppressed lattice distortions caused by Mn^3+^ and inhibited gliding of the TM layer at high charged states and thus improved performance. Though the solid‐state reaction route is straightforward and suitable for mass production, tuning the particle size and morphology of the active materials present significant challenges. As the alkali and transition metal ions diffuse through solid phases to occupy their respective atomic positions, this method often requires time‐consuming, high‐temperature calcination to obtain the desired crystal structure without any impurities. Further, the physical mixing of precursors results in a nonuniform product with impurities and thus researchers have largely adopted solution‐based methods that enable cation mixing at the atomic level and the formation of homogenous precursors in chemically controlled conditions.^[^ ^132^ ^]^

### 4.2 Solvothermal/Hydrothermal Methods

Solvothermal/hydrothermal methods are facile and environmentally benign processes because the reactions are carried out in closed containers. The hydrothermal method uses an aqueous solvent whereas the solvothermal method uses organic solvents. Yuan et al.^[^ ^68^ ^]^ synthesized a K_0.486_V_2_O_5_ cathode via hydrothermal‐assisted heat treatment. Typically, K_2_CO_3_ and V_2_O_5_ were dissolved in water containing a reducing agent (30% H_2_O_2_). The solution was heated in an autoclave 180 ° C for 3 h. After cooling, the precipitate was washed with water and ethanol and finally heated at 450 ° C for 2 h. The SEM image of K_0.486_V_2_O_5_ cathode, which exhibited a high specific capacity of 159 mAh g^−1^ at 20 mA g^−1^, revealed a nanobelt morphology. Hydrothermal/solvothermal methods can achieve various morphologies and particle sizes by tuning the reaction conditions and reducing agents. Liu et al.^[^ ^69^ ^]^ obtained layered K_0.23_V_2_O_5_ with a flower‐like morphology, which exhibited enhanced electrochemical performance owing to its multilayered microstructure which offered more void space to accommodate volume changes caused by K^+^ insertion/desertion. Deng et al.^[^ ^44^ ^]^ synthesized K_0.65_Fe_0.5_Mn_0.5_O_2_ cathodes by two different routes (viz. hydrothermal and conventional solid‐state reactions) to demonstrate the influence of the synthetic procedure on the properties of the cathode. Initially, a (Fe_0.5_Mn_0.5_)_2_O_3_ precursor with homogeneously mixed Fe and Mn at the atomic level was obtained by hydrothermal reaction followed by heat treatment. (Fe_0.5_Mn_0.5_)_2_O_3_ was then mixed with KOH before the mixture was finally calcined at 1000 ° C for 12 h to yield s‐K_0.65_Fe_0.5_Mn_0.5_O_2_ microspheres. Conversely, the c‐K_0.65_Fe_0.5_Mn_0.5_O_2_ synthesized through the solid‐state route by physical mixing and calcined under the same conditions produced irregularly‐shaped particles. The s‐K_0.65_Fe_0.5_Mn_0.5_O_2_ microspheres exhibited superior capacity and durability to the irregularly shaped c‐K_0.65_Fe_0.5_Mn_0.5_O_2_ particles. Further, the irregular particles pulverized and formed an unstable passivation layer while the microspheres formed a uniform interface between the cathode and electrolyte and thus inhibited side reactions during cycling. In another work, ternary P3‐K_0.5_Mn_0.72_Ni_0.15_Co_0.13_O_2_ microspheres were achieved by solvothermal method.^[^ ^98^ ^]^ Secondary microspheres composed of sub‐micron‐sized primary particles minimize the diffusion distance of K^+^ ions and provide structural stability by acting as buffer during K^+^ extraction/insertion. Interestingly, P3‐K_0.48_Ni_0.2_Co_0.2_Mn_0.6_O_2_ with a mixed morphology containing microspheres and microcubes was achieved by a solvothermal method using glycerol and urea additives.^[^ ^112^ ^]^ Though hydrothermal/solvothermal methods provide highly crystalline products with tailored morphologies and uniform particle distributions, their use is limited owing to the necessity of using high‐pressure vessels and their comparatively low product yields.

### 4.3 Sol–Gel

The sol–gel method is a wet chemical process involving the formation of a "sol" by polymerization of metal salts in a solvent and subsequent transformation of the sol into a porous "gel" network of colloidal particles. Finally, dehydration and heat treatment of the sol–gel yields powders with the desired crystallinity.^[^ ^126^ ^]^ The chelating agent and solution pH are important parameters in controlling the particle size, porosity, and morphology of the products.^[^ ^134^ ^]^ The sol–gel method operates at lower processing temperatures and achieves more uniform mixing of metal precursors than conventional state‐state methods. Ramasamy et al.^[^ ^81^ ^]^ prepared P3‐K_0.45_Mn_0.5_Co_0.5_O_2_ nanoplatelets via a sol–gel process involving metal acetate precursors and a citric acid chelating agent followed by sintering at 800 ° C. Lei et al.^[^ ^53^ ^]^ employed a sol‐gel method to fabricate a P2‐K_0.67_MnO_2_ cathode which delivered a discharge capacity of 78 mAh g^−1^ at 50 mA g^−1^ and exhibited high cyclic stability, retaining 90% of its initial capacity after 300 cycles. Liu et al.^[^ ^105^ ^]^ achieved multielement doping via a citric acid‐assisted sol–gel process followed by calcination at high temperature. The synthesized P3‐K_1/2_Mn_5/6_Mg_1/12_Ni_1/12_O_2_ showed a uniform distribution of smaller particles which reduced the K^+^ ion transport distances. In the sol–gel, the pH of the solution must be controlled to prevent the sol from precipitating rather than forming a gel. Additionally, controlling the particle size during high‐temperature heat treatment by the selection of precursors and reaction conditions is challenging.

### 4.4 Coprecipitation

The coprecipitation is a solution method, which can produce a precipitate consisting of uniformly mixed metal ions with the aid of a precipitating agent. The precipitate is then mixed with a source of alkali metal and the mixture is annealed at an elevated temperature to obtain the target compound.^[^ ^128^ ^]^ Liu et al. fabricated P2‐K_0.3_MnO_2_ and P3‐K_0.45_MnO_2_ cathodes by coprecipitation‐assisted calcination.^[^ ^46^ ^]^ Aqueous metal acetate solution was mixed with oxalic acid (precipitant) under stirring and the filtered precipitate was calcined at 900 ° C for 15 h. yielding the active materials. The smaller P3‐K_0.45_MnO_2_ particles result in a cathode with superior performance to that of the P2‐K_0.3_MnO_2_. In the co‐precipitation process, the solution pH, temperature, and concentration can be controlled to achieve a homogeneous particle distribution and the required morphology. Peng et al.^[^ ^48^ ^]^ synthesized hollow P3‐K_0.5_MnO_2_ nanospheres with average diameter of 600 nm by controlling the parameters of the coprecipitation reaction. The synergistic effect of the spherical morphology and interior voids provided the P3‐K_0.5_MnO_2_ cathode with a high volumetric energy storage capacity and cycling stability. Liu et al.^[^ ^97^ ^]^ prepared a K_0.67_Ni_0.17_Co_0.17_Mn_0.66_O_2_ cathode via coprecipitation followed by a solid‐state reaction. The crystallinity of the powder was found to increase with the increasing sintering temperature. Post heat treatment is necessary to improve the crystallinity of compounds synthesized by coprecipitation methods. Further, the precipitation agent, pH, stirring speed, and temperature must be carefully adjusted to ensure the quality of the products.

### 4.5 Combustion

Combustion is used to produce a finely mixed precursor or the desired active materials by spontaneous combustion between the reactants.^[^ ^135^ ^]^ The desired product may be obtained by further heating the homogenous precursor mixture produced by the combustion process. Choi et al.^[^ ^82^ ^]^ reported the preparation of K_0.54_[Co_0.5_Mn_0.5_]O_2_ powder by a combustion method. Metal nitrates and citric acid (chelating agent) were first dissolved in water, and the aqueous solution was heated at 200 ° C to initiate autocombustion of the citric acid. The resultant powder was calcined at 800 ° C for 5 h to obtain the target compound. Cho et al.^[^ ^76^ ^]^ synthesized P3‐K_0.5_[Ni*_x_* Mn_1‐_ *_x_*]O_2_ (*x* = 0 and 0.1) via the combustion of metal nitrates, using citric acid and sucrose as a chelating agent and agglomeration inhibitor, respectively. The P3‐K_0.5_[Ni_0.1_Mn_0.9_]O_2_ cathode showed superior K^+^ storage capacity and structural stability to the pristine cathode. Although combustion is a simple and facile process, it is a highly exothermic reaction and produces potentially toxic fumes, imposing significant health and safety risks.

### 4.6 Electrochemical Ion Exchange

The ion‐exchange method is an efficient strategy for the synthesis of metastable layered metal oxide compounds.^[^ ^135^, ^136^ ^]^ The ion‐exchange process replaces the mobile ions in the host material with guest ions, which enables the production of novel intercalation compounds that cannot be synthesized by conventional heat treatment.^[^ ^129^ ^]^ This strategy is largely used to prepare LIB and SIB cathodes by ion exchange in solutions or molten salts of Li and Na.^[^ ^128^, ^137^ ^]^ in electrochemical ion exchange, the ion exchange of the intercalation cathode is carried out in an electrochemical cell. The host cathode is typically charged/discharged in an electrolyte containing the target species to obtain the desired compound.^[^ ^129^ ^]^ The solid‐state synthesis of layered metal oxide compounds with high K^+^ content is challenging because the large size of K^+^ ions causes strong K^+^–K^+^ repulsion and thermodynamically unstable conditions.^[^ ^40^ ^]^ However, electrochemical ion‐exchange method shows great promise for the synthesis of K^+^‐layered cathodes with desired compositions. Hwang et al.^[^ ^66^ ^]^ reported the synthesis of P3‐K_0.69_CrO_2_ via electrochemical ion exchange of an O3‐NaCrO_2_ sodium compound. O3‐NaCrO_2_ was first synthesized by solid‐state method, then the NaCrO_2_ cathode was galvanostatically cycled in a cell with the configuration K‐metal | 0.5 m KPF_6_ in EC:DEC | NaCrO_2_. The Na^+^ ions are gradually substituted with K^+^ ions, yielding P3‐K_0.69_CrO_2_ after 300 cycles. The solvation energy of Na^+^ is lower than that of K^+^ in organic electrolytes, which inhibits the ion‐exchange process and necessitates several cycles to produce P3‐K_0.69_CrO_2_ without Na^+^ content. The P2‐K_2/3_Ni_1/3_Mn_2/3_O_2_ cathode fabricated from P2‐Na_2/3_Ni_1/3_Mn_2/3_O_2_ via electrochemical ion‐exchange showed fast chargeable characteristics.^[^ ^41^ ^]^ Using a similar strategy, Myung et al. developed P2‐K_0.75_[Ni_1/3_Mn_2/3_]O_2_ and P’2‐K_0.83_[Ni_0.05_Mn_0.95_]O_2_ electrodes with higher K^+^ content, which demonstrated improved capacity and structural stability without undergoing P2‐OP_4_ phase transitions.^[^ ^84^, ^88^ ^]^ A fuel cell with a P2‐K_0.75_[Mn_0.8_Ni_0.1_Fe_0.1_]O_2_ cathode obtained by ion exchange and a hard carbon anode delivered a charge capacity of 60 mAh g^−1^ and retained 60% of its capacity over 1000 cycles. Nathan et al.^[^ ^12^ ^]^ compared the electrochemical properties P2‐K_0.70_[Cr_0.85_Sb_0.15_]O_2_ cathodes prepared by solid‐state and electrochemical ion‐exchange methods. The residual Na^+^ present in the P2‐K_0.62_Na_0.08_[Cr_0.85_Sb_0.15_]O_2_ (IE‐KCSO) cathode even after a long exchange reaction gives the IE‐KCSO a superior rate capability with smaller dimensional changes than the cathode synthesized by the solid‐state method. However, further investigations are required to fully understand the complex ion‐exchange reaction mechanisms. Electrochemical ion exchange can produce novel K^+^‐layered cathodes with improved properties; however, its commercial viability is limited by its the time‐consuming, multistep procedure.

## 5 Challenges and Strategies

This section will summarize the applications, electrochemical performance, and structural changes in K^+^‐based layered metal oxide cathodes for applications in KIBs. Layered metal oxides with long K^+^ diffusion paths and high capacities are promising cathode materials for KIBs, which may be suitable substitutes for LIBs owing to the natural abundance of potassium, the reversible nature of K^+^ intercalation into the graphite anode, higher K^+^ mobility and compatibility with the economical aluminum current collector. However, suitable cathode materials are crucial for realizing practical KIBs. Although existing layered metal oxide cathodes demonstrate acceptable electrochemical performance in KIBs, the low K^+^ content and air instability of the synthesized materials, and the multiple structural changes that occur during K^+^ extraction/insertion result in inferior cycling stability. Many strategies have been employed to address these issues and enhance the performance of layered metal oxide cathodes, including element doping, surface coating, and tailoring of their structure and morphology.

### 5.1 Air Instability

Layered metal oxide cathodes typically suffer from instability in ambient conditions owing to their hygroscopic nature. The layered cathode materials rapidly adsorb water or CO_2_ molecules upon exposure to air/moisture, causing significant structural deformation and deterioration in cyclic stability. The Ni‐rich layered cathodes LiNi*_x_* Co*_y_* Mn*_z_* O_2_ (NCM) and LiNi*_x_* CoyAl*_z_* O_2_ (NCA) employed in practical LIBs also encounter these issues. The chemically sensitive Ni‐rich surface reacts with moisture and CO_2_ to form residual lithium compounds such as LiOH and Li_2_CO_3_, which impose significant safety risks.^[^ ^6^, ^95^ ^]^ Na^+^‐based layered oxide cathodes undergo a similar reaction upon exposure to air, forming NaOH and Na_2_CO_3_ compounds on the surface of the active materials. These residual insulating and electrochemically inert compounds degrade the electrode performance.^[^ ^138^ ^]^ K^+^‐based layered cathodes are more vulnerable to air/moisture than Li^+^/Na^+^ layered cathodes owing to the larger interlayer spacing in K^+^‐containing cathodes, which more easily accommodate H_2_O/CO_2_ molecules. This type of cathode must therefore be meticulously stored and handled in a moisture‐free/inert atmosphere, increasing their production and transportation costs. Single‐metal K*_x_* MO_2_ compounds may be especially prone to air instability. For instance, XRD patterns of P2‐K_0.41_CoO_2_ exposed to humid air for 24 h showed additional peaks corresponding to the hydrated phase formed by the absorption of water molecules.^[^ ^43^ ^]^ After drying at 150 ° C, these additional peaks disappeared but the P2‐K_0.41_CoO_2_ structure showed different lattice parameter values than the pristine compound. The adsorbed water may cause an undesirable reaction between the K‐metal anode and the electrolyte. Despite the large number of hygroscopic K^+^‐layered cathodes, strategies for improving their air stability are limited, especially in comparison to those for improving the air stability of Na^+^‐layered cathodes.^[^ ^138^ ^]^ K^+^‐layered cathodes are typically handled in an Ar‐filled glovebox to minimize the influence of moisture. During XRD measurements, the sample is protected from exposure to air by covering the sample holder covered Kapton film. Moreover, slurries and electrodes containing K^+^‐layered materials are also made in a glovebox.^[^ ^29^, ^44^, ^56^, ^74^, ^94^, ^101^, ^103^ ^]^ Synthetic routes to air‐stable K^+^‐layered compounds are therefore critical for the realization of practical KIBs. Research concerning the development of KIBs is still in its infancy; however, taking cues from Li^+^/Na^+^ layered cathodes is a fair approach to the design of air‐stable K^+^‐layered cathodes.

Air‐stable Na^+^‐layered cathodes have recently been developed for application in practical NIBs. In addition to the well‐known air‐stable P2‐Na_2/3_Ni_1/3_Mn_2/3_O_2_,^[^ ^139^ ^]^ other air‐stable cathodes have been developed from different polymorphs such as P2‐Na_7/9_Cu_2/9_Fe_1/9_Mn_2/3_O_2_,^[^ ^140^ ^]^ P3‐Na_2/3_Ni_1/4_Mg_1/12_Mn_2/3_O_2,_^[^ ^141^ ^]^ and O3‐Na[Li_0.05_Mn_0.50_Ni_0.30_Cu_0.10_Mg_0.05_]O_2_.^[^ ^142^ ^]^ These materials retain their original structure even after soaking in water for several days and thus demonstrate excellent air/moisture stability. Mu et al.^[^ ^143^ ^]^ reported air‐stable and Co/Ni‐free O3‐Na_0.9_[Cu_0.22_Fe_0.30_Mn_0.48_]O_2_ in which Cu^2+^/Cu^3+^ and Fe^3+^/Fe^4+^ redox couples take part in a charge compensation mechanism. These studies indicate that the substitution of divalent metal ions (Cu^2+^/Mg^2+^) can efficiently improve the stability of these layered cathodes to moisture. Yao et al.^[^ ^144^ ^]^ improved air stability by increasing the valance state of the transition metals while simultaneously decreasing the Na^+^ interlayer spacing, which they termed a “combined structure modulation.” They designed a O3‐NaNi_0.45_Cu_0.05_Mn_0.4_Ti_0.1_O_2_ material by co‐doping Cu^2+^ Ti^4+^, which have comparable electronegativities but substantially different Fermi levels. The Cu/Ti co‐doped cathode demonstrated a stable air‐exposure period and capacity retention after 500 cycles 20 times and 9 times greater, respectively than the undoped cathode.

Surface coating strategy can also improve the stability of layered cathode materials under ambient conditions. Manthiram et al. improved the chemical stability of O3‐layered oxide cathodes to moisture using various strategies, including modifying the cathode surface with a ZrO_2_ coating.^[^ ^145^ ^]^ Incorporating LiF into the crystal structure of the cathode introduces Li^+^ ions that suppress the Na^+^/H^3^O^+^ exchange process while the formation of strong oxyfluoride bonds stabilizes the host structure and prevents the release of lattice oxygen upon contact with water.^[^ ^146^ ^]^ Some vanadium‐based compounds are reported to improve the air stability of K^+^‐layered cathodes. Zhang et al.^[^ ^74^ ^]^ developed a moisture‐stable layered K_0.83_V_2_O_5_ material in which K^+^ is sandwiched between puckered [V_2_O_5_] layers. The preinsertion of water into crystal lattice of layered cathodes could adjust the interlayer spacing and thereby enhance the stability of the host structure. For instance, a layered K_0.28_MnO_2_·0.15H_2_O cathode exhibited a discharge capacity of 150 mAh g^−1^ and high stability over 100 cycles.^[^ ^28^ ^]^ Similarly, K_0.27_Mn_0.98_O_2_·0.53H_2_O demonstrated superior cyclic stability to K*_x_* MnO_2_ compounds.^[^ ^65^ ^]^ The high stability of these materials arises from the presence of crystal water that serve as pillars during K^+^ extraction/insertion, providing structural stability and an enlarged interlayer space and thus facilitating faster K^+^ diffusion. However, crystal water may decompose at voltages over 4 V versus K/K^+^, which may reduce capacity and induce side reactions that may cause safety problems. Therefore, optimizing the crystal water content is crucial because the number of K^+^ storage sites may be limited by excess water.

In summary, Na^+^‐layered cathodes with improved air stability have been achieved using structure modulation and surface coating strategies. The stability of the cathodes can be improved by the doping of electrochemically active (Cu^2+^) or inactive (Mg^2+^) ions. In particular, electrochemically active dopants participate in the storage of alkali ions via the charge compensation mechanism. The substitution of Ti^4+^ increases the average valance state of the transition metals and alters the interlayer spacing between the alkali metal layers, thereby inhibiting the insertion of water molecules. Coating the surface of the materials with carbon enhances their chemical stability and conductivity, and also works as a protective layer to suppress the degradation of the cathode materials. The strategies used to develop air‐stable Li^+^/Na^+^ layered cathodes offer insights into the design of K^+^‐layered cathodes with enhanced stability. The electronegativity of the metal dopant is a key parameter in the rational design of cathode materials. Electrochemically active (Cu, Ni, Fe, Mn) and inactive (Mg, Zn, Al, Ti) metals may be substituted into the layered cathodes to enhance their stability. In addition to the reported metal oxides, protective coatings can be formed using other metal oxides such as Al_2_O_3_, TiO_2_, ZnO, and MgO. The development of KIBs is in an emerging stage and thus further investigation is required to identify suitable metal dopant and coating materials to improve the stability of K^+^‐layered cathodes.

### 5.2 K^+^‐Deficient Compounds

Most existing K^+^‐layered metal oxide cathodes consist of nonstoichiometric compounds. Computational studies reveal that the formation of K^+^‐deficient layered cathodes is caused by strong K^+^–K^+^ interactions.^[^ ^40^ ^]^ The K^+^‐ions preferentially occupy large prismatic sites rather than smaller octahedral sites owing to their size, and thus most of the compounds stabilize in K^+^‐deficient P2 or P3 phases. The low K^+^ content of the synthesized layered cathode materials reduces the specific capacity of the full cell because the cathode material is a reservoir of K^+^‐ions since the K‐metal anode cannot be used owing to safety risks. The charge capacity of these K^+^‐deficient cathodes is lower than their discharge capacity during the initial charge/discharge process, resulting in a coulombic efficiency greater than 100% (Figure 9a): however, the K^+^‐ions are reversibly accessible from the second cycle.^[^ ^47^, ^48^, ^53^, ^56^, ^82^ ^]^ This behavior limits the specific capacity and energy density of the full cell constructed with a carbon‐based anode, which lacks a source of K^+^. Accordingly, strategies to develop layered cathode materials with a high K^+^‐content are needed to obtain improve the electrochemical performance of the cathodes. Kim et al.^[^ ^40^ ^]^ synthesized a stoichiometric O3‐KCrO_2_ cathode that delivered a discharge capacity of 92 mAh g^−1^; however, this material requires further optimization owing to its multiple phase transitions and low cyclic stability. The incorporation of large cations such as Sc^3+^ and Y^3+^ may stabilize O3‐K*_x_* MO_2_ layered structures. Moreover, the crystal structure and morphology of host materials are significantly influenced by the higher K^+^ content in layered cathodes. A P3‐K_0.45_MnO_2_ cathode with a high K^+^ content exhibited a smaller particle size, improved cyclic stability, and superior rate performance to P2‐K_0.3_MnO_2_.^[^ ^46^ ^]^ Lin et al. prepared a series of birnessite nanosheet arrays with varying K content (K_x_MnO_2_·yH_2_O) by a hydrothermal potassiation process.^[^ ^147^ ^]^ The K_0.77_MnO_2_·0.23H_2_O birnessite with an optimized K^+^ content delivered a high specific capacity of 134 mAh g^−1^ at 100 mA g^−1^ and excellent cycling stability with 80.5% capacity retention after 1000 cycles at 1000 mA g^−1^. Notably, enough K^+^ was retained in the cathode in the charged state to stabilize the layered structure and mitigate the structural degradation that occurs during K^+^ extraction/insertion. K^+^‐layered cathode materials with improved K^+^ content can be efficiently synthesized by electrochemical ion exchange. Layered cathodes such as P2‐K_2/3_[Ni_1/3_Mn_2/3_]O_2_,^[^ ^41^ ^]^ P2‐K_0.75_[Ni_1/3_Mn_2/3_]O_2_,^[^ ^84^ ^]^ P′2‐K_0.83_[Ni_0.05_Mn_0.95_]O_2_^[^ ^88^ ^]^ and P2‐K_0.75_[Mn_0.8_Ni_0.1_Fe_0.1_]O_2_^[^ ^100^ ^]^ were fabricated via electrochemical ion exchange using various Na^+^‐layered cathodes as templates. The carbon‐based anodes are sometimes prepotassiated to compensate for the K^+^ deficiency of the cathodes in full cell KIBs; however, this additional process may increase the production cost.^[^ ^45^, ^71^, ^90^ ^]^ Sacrificial salts are used in Na^+^‐deficient layered cathodes to circumvent the irreversible capacity in the first cycle. For instance, sacrificial NaN_3_ salts are added to P2‐Na_2/3_[Fe_1/2_Mn_1/2_]O_2_,^[^ ^148^ ^]^ Na_3_P for P2‐Na_0.67_[Fe_0.5_Mn_0.5_]O_2_,^[^ ^149^ ^]^ NaNO_2_ for P′2‐Na_2/3_[Co_0.05_Mn_0.95_]O_2_^[^ ^150^ ^]^ and ethylenediaminetetraacetic acid (EDTA) tetrasodium salt is added to P2‐Na_2/3_[Al_0.05_Mn_0.95_]O_2_^[^ ^151^ ^]^ to serve as an additional Na^+^ source and improve the initial coulombic efficiencies of the materials. However, the gas generated by the decomposition of these additive salts during cycling and the energy penalty issues remain to be addressed. Inspired by Na^+^‐layered cathodes, research efforts are required to identify suitable sacrificial additives for K^+^ deficiency in the pristine K^+^‐layered cathodes.

### 5.3 Steeper Voltage Profiles

The voltage profiles of K*_x_* MO_2_ layered cathodes typically exhibit steeper slopes than those of Li^+^ and Na^+^ layered compounds. O2‐LiCoO_2_ demonstrated narrow range reversible Li^+^ deintercalation/intercalation with a flat voltage profile with fewer voltage steps (Figure 5a); however, P2‐Na_2/3_CoO_2_ exhibited more distinct voltage plateaus with a steeper voltage curve, and this phenomenon is more prominent in P2‐K_0.41_CoO_2_.^[^ ^43^ ^]^ This behavior is mainly related to the large size of the K^+^ ions, which increases the interlayer spacings and thus reduces the screening of K^+^–K^+^ repulsion by adjacent oxygen layers. The strong K^+^–K^+^ interactions induce K^+^/vacancy ordering and transition metal charge ordering, resulting in multiple phase transitions and the associated stepwise voltage curves.^[^ ^20^, ^32^, ^38^, ^90^ ^]^ The phase transitions between these K^+^/vacancy ordered configurations limit the K^+^ diffusion kinetics and a number of K^+^ storage sites, reducing the achievable capacity in a given voltage window. Accordingly, layered K*_x_* MO_2_ compounds always deliver lower discharge capacities than their Li*_x_* MO_2_ and Na*_x_* MO_2_ counterparts. However, studies concerning K^+^/vacancy ordering are comparatively rare in relation to those on Na^+^/vacancy ordering in Na^+^‐layered cathodes. Three related types of ordering occur in layered cathodes viz., transition metal ordering, charge ordering, and K^+^/vacancy ordering. Neither K^+^/vacancy ordering nor charge ordering can exist without the other. The large difference between the ionic radii of the transition metal ions induces metal ordering, while the smaller difference between the redox potentials of the metal ions induces charge ordering.^[^ ^90^, ^152^, ^153^ ^]^ Therefore, suppressing K^+^/vacancy ordering to produce a disordered K^+^/vacancy structure is important to enhance battery performance. Wang et al.^[^ ^154^ ^]^ developed cation‐disordered P2‐Na_0.6_[Cr_0.6_Ti_0.4_]O_2_ by doping with Cr^3+^ and Ti^4+^, which have similar ionic radii but different redox potentials. P2‐Na_0.6_[Cr_0.6_Ti_0.4_]O_2_ showed a completely disordered Na^+^/vacancy structure regardless of the sodium content and demonstrated enhanced cyclic stability and rate capability. Similarly, partial substitution of Ti^4+^ with Mn^4+^, which has a compatible ionic radius but different Fermi level, in P2‐Na_2/3_Ni_1/3_Mn_1/3_Ti_1/3_O_2_ suppressed both the Na^+^/vacancy ordering and charge ordering, resulting in a smoother voltage profile and superior performance to pristine P2‐Na_2/3_Ni_1/3_Mn_2/3_O_2_ cathode.^[^ ^155^ ^]^ Mao et al. adopted an anion/cation (CaF_2_) dual doping strategy to synthesize Na_2/3_Ni_1/3_Mn_2/3_O_2_.^[^ ^156^ ^]^ The inactive Ca^2+^ doped into the Na^+^‐layer mitigates the Na^+^/vacancy ordering and acts as a pillar to stabilize the layer structure of the highly desodiated state. The more electronegative F^−^ ion forms a stronger bond with transition metal ions, thereby reducing the interactions between the oxygen layers. Codoping with Ca^2+^ and F^−^ suppressed the P2‐O2 transition and improved conductivity for high‐rate capability. Ramasamy et al.^[^ ^81^ ^]^ reported a binary metal layered K_0.45_Mn_0.5_Co_0.5_O_2_ cathode that showed smoother K^+^ extraction/insertion lower stepwise voltages (Figure 9a) than single‐metal cathodes such as K*_x_* MnO_2_ and K*_x_* CoO_2_.^[^ ^46^, ^56^ ^]^ The content and distribution of K^+^ in the K^+^‐layer alter the K^+^–K^+^ repulsion interactions and transition metal–oxygen bonds. In addition to this cation doping strategy, Xiao et al.^[^ ^90^ ^]^ developed K^+^/vacancy disordered K_0.7_Mn_0.7_Ni_0.3_O_2_ by modulating the K^+^ content of the compound. Stable K^+^/vacancy disordered K*_x_* Mn_0.7_Ni_0.3_O_2_ (*x* = 0.4–0.7) compounds formed when *x* > 0.6. The capacity and rate performance of K^+^/vacancy disordered K_0.7_Mn_0.7_Ni_0.3_O_2_ were superior to those of K^+^/vacancy ordered K_0.4_Mn_0.7_Ni_0.3_O_2_.

In summary, the K^+^/vacancy ordering related phase transitions and steep voltage curves limit the capacity and life cycle of practical batteries. Therefore, different strategies to form disordered structures and mitigate K^+^/vacancy ordering are vital. The substitution of cations (Mg^2+^, Zn^2+^, Ti^4+^, Sb^5+^) with ions of comparable ionic radii and different redox potentials produces K^+^/vacancy disordered compounds that may exhibit smoother voltage profiles and improved cyclability and rate capability. K^+^/vacancy disordered compounds can also be efficiently formed by modulating the K^+^ content of the layered structure. In addition, suitable strategies to suppress the K^+^/vacancy ordering are required.

### 5.4 Irreversible Structural Changes

Layered metal oxide cathodes commonly undergo a series of structural transitions and volume changes as a result of K^+^/vacancy ordering and sliding of transition metal oxide (TMO_2_) slabs during K^+^ extraction/insertion. When a certain amount of K^+^ is extracted, the strong K^+^–K^+^ repulsion interactions induce the gliding of adjacent oxygen layers. Further, extreme depotassiation causes irreversible phase transitions such as P2‐O2 and P3‐O3 in P2‐type P3‐type materials, respectively. These irreversible phase changes cause rapid capacity fading and structural failure of the layered cathode materials.^[^ ^29^, ^41^, ^42^, ^45^, ^47^ ^]^ Kim et al.^[^ ^47^ ^]^ reported a P3‐K_0.5_MnO_2_ cathode which underwent multiple phase transitions from P3 to an O3 phase and an unknown phase (X phase) during K^+^ extraction/insertion, resulting in rapid capacity degradation (Figure 3c). The upper cutoff voltage is typically limited to circumvent the irreversible phase transition in the deep depotassiated state; however, this reduces the maximum achievable capacity and average voltage of the batteries.^[^ ^41^, ^45^, ^153^ ^]^ Therefore, irreversible phase changes must be avoided to ensure optimum battery performance at high voltage. The Jahn‐Teller (J‐T) effect also causes structural degradation and capacity fading in K*_x_* MnO_2_ layered cathodes. The presence of high spin Mn^3+^ (3d^4^) in the K*_x_* MnO_2_ layered structure elongates the Mn—O bond along one direction in MO_6_ octahedron in the discharged state (Figure 9e). This J‐T effect induces severe lattice distortions in the crystal structure that lead to performance degradation and ultimately structural collapse.^[^ ^53^, ^82^ ^]^ Metal doping can efficiently mitigate these structural distortions, resulting in smoother electrochemistry in Li*_x_* MnO_2_ and Na*_x_* MnO_2_ compounds containing Mn^3+^.^[^ ^157^, ^158^ ^]^ However, because the larger K^+^ ions exhibit different electrochemical behaviors and structural evolutions, a more thorough understanding of these strategies and their effects in KIB materials is required. Moreover, material design strategies to address these issues and improve the structural stability, reversible capacity, electronic/ionic conductivity, long‐term cyclability, average voltage, and energy density of electrode materials for application in KIBs are highly desirable.

### 5.5 Element Substitution

**Figure 15:**
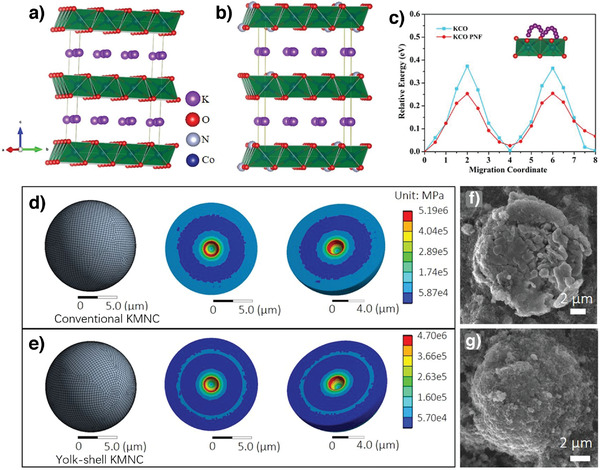
Crystal structures of a) K_0.6_CoO_2_ (KCO), b) K_0.6_CoO_1.8_N_0.2_ porous nanoframe (KCO PNF) and c) K^+^ migration energy barriers of KCO and KCO PNF. Reproduced with permission.^[^ ^159^ ^]^ Copyright 2020, Elsevier. d,e) Finite element model of strain variation during volume expansion and f,g) SEM images of conventional KMNC and YS‐KMNC after cycling. Reproduced with permission.^[^ ^162^ ^]^ Copyright 2022, Wiley.

Element substitution shows great promise as a strategy to mitigate the irreversible phase transitions and enhance the structural stability and electrochemical performance of K^+^‐layered oxide materials. The partial substitution of Ni^2+^ for Mn^3+^ in K*_x_* MnO_2_ compounds effectively reduces J‐T distortions and stabilizes the crystal structure, thereby enhancing electrochemical performance.^[^ ^76^, ^77^, ^89^ ^]^ Interestingly, both theoretical and experimental studies demonstrate that P2‐K_0.75_[Ni_1/3_Mn_2/3_]O_2_ undergoes a single‐phase reaction involving a Ni^4+^/Ni^2+^ redox couple while Mn^4+^ is inactive during charge/discharge in the operation range.^[^ ^84^ ^]^ The P2‐K_0.75_[Ni_1/3_Mn_2/3_]O_2_ delivered a discharge capacity of 91 mAh g^−1^ and showed excellent cycling stability with 83% capacity retention after 500 cycles at high current rate of 1400 mA g^−1^. Choi et al.^[^ ^88^ ^]^ developed P’2‐K_0.83_[Ni_0.05_Mn_0.95_]O_2_ by retaining the Pʹ2 phase without transitioning to OP4 during K^+^ extraction/insertion, which showed excellent cyclability. First‐principles calculations revealed that a low activation energy barrier (271 meV) leads to high power capability by enabling facile K^+^ diffusion in P’2‐K_0.83_[Ni_0.05_Mn_0.95_]O_2_. Zhang et al.^[^ ^75^ ^]^ demonstrated that small amount of Co (5%) incorporated at Mn sites in K*_x_* MnO_2_ reduced J‐T distortions and improved ionic conductivity, as well as facilitated more isotropic migration pathways for K+ diffusion in the interlayer structure, leading to enhanced ion diffusion and rate capability. The (*a/b*) ratio of K*_x_* Mn_0.95_Co_0.05_O_2_ is lower than that of undoped K*_x_* MnO_2_, confirming that the lattice distortions were effectively reduced. Layered K*_x_* MO_2_ commonly exhibits sluggish K^+^ diffusion and multistep voltage plateaus upon K^+^ deintercalation/intercalation. However, introducing Co into P3‐K_0.54_[Co_0.5_Mn_0.5_]O_2_ resulted in smoother charge/discharge curves and enhanced rate performance.^[^ ^82^ ^]^ This may be attributed to the incorporation of Co, which minimized structural distortions and reduced the K^+^ diffusion barrier. Xu et al.^[^ ^29^ ^]^ prepared K_5/9_Mn_7/9_Ti_2/9_O_2_ by substituting Mn^4+^ with Ti^4+^, which has a similar valence state and comparable ionic radius. Unlike undoped K_5/9_MnO_2_, K_5/9_Mn_7/9_Ti_2/9_O_2_ underwent a reversible P2‐OP4 phase transition, thus preventing the formation of the destructive P2‐O2 phase (Figure 11e,f). The existence of Ti^4+^ significantly reduced the gliding of TMO_2_ slabs during the charge/discharge process. Despite the specific capacity loss caused by the electrochemically inactive Ti^4+^, it minimized the step‐like voltage transitions and thus improved the electrochemical behavior. Zhang et al.^[^ ^102^ ^]^ developed a P3‐type K_0.4_Fe_0.1_Mn_0.8_Ti_0.1_O_2_ electrode by incorporating inexpensive and environmentally friendly Fe and Ti. This electrode showed a rapid rate capability (71 mAh g^−1^ at 1000 mA g^−1^) and excellent cycling stability over 300 cycles with a negligible volume change of 0.5% upon K^+^ extraction/insertion, which was attributed to the suppression of multiple irreversible phase transitions originating from the presence of inactive Ti^4+^ and strong Ti—O bonds relative to Fe/Mn—O bonds. The stronger Ti—O bonds may increase the number of Mn—O and Fe—O covalencies and thus suppress the movement of (Mn, Fe)O_6_ octahedra by sharing oxygen atoms with Ti, resulting in structural stability and high‐rate performance.^[^ ^102^ ^]^ Dang et al. studied the influence of Mg^2+^ and Al^3+^ substitution in P3‐type K_0.45_Ni_0.1_Co_0.1_Mn_0.8_O_2_.^[^ ^99^ ^]^ The Mg/Al‐doped compounds exhibited superior cycling and rate performance to the pristine compound owing to their displayed enlarged K^+^ diffusion layers and reduced J‐T distortions. Moreover, the J‐T distortions were more heavily suppressed in the Mg‐doped compound than in the Al‐doped compound. Nathan et al.^[^ ^12^ ^]^ prepared inactive Sb^5+^ substituted P2‐K_0.70_Cr_0.85_Sb_0.15_O_2_ via a solid‐state reaction. The inductive effect of Sb^5+^ increased the average voltage (2.9 V) relative to that of O3‐KCrO_2_ and P’3‐K_0.8_CrO_2_ (2.7 V).^[^ ^40^, ^42^ ^]^ Liu et al. reported that Na doping in K_0.67_Ni_0.17_Co_0.17_Mn_0.66_O_2_ stabilized the layered structure and improved the redox reaction of transition metal ions, resulting in improved electrochemical performance.^[^ ^94^ ^]^ Yu et al.^[^ ^159^ ^]^ took a different approach to these cation doping strategies by developing a porous K_0.6_CoO_2‐_ *_x_* N*_x_* nanoframe by incorporating nitrogen (N) in the anion site (**Figure** **15**a,b). The K_0.6_CoO_1.8_N_0.2_ cathode delivered a discharge capacity of 86 mAh g^−1^ at 50 mA g^−1^ and retained 77.3% of its capacity after 400 cycles. Figure 15c compares the K^+^ migration energy barriers of K_0.6_CoO_2_ and K_0.6_CoO_1.8_N_0.2_. Experimental and computational studies reveal that the partial substitution of O with N increases both the interlayer spacing and electronic conductivity and thus improves long‐term cyclability and rate capability.

These studies reveal that the substitution of metal elements such as Ni, Co, Fe, Ti, Mg, and Al in layered K*_x_* MnO_2_ suppresses J‐T distortions and structural changes during K^+^ deintercalation/intercalation. The design of electrode materials containing low‐cost, and environment‐friendly elements such as Mn, Fe, etc., is highly preferable with regard to the development of practical KIBs.

### 5.6 Morphology Design

Morphology design can effectively improve a material's high‐rate capability and durable cycling performance. The fabrication of micro/nanostructured particles shortens the K^+^ diffusion pathway and improves the conductivity. However, nanoparticles may cause low tap density owing to their large specific area and side reactions with electrolytes; further study is required to address these issues.^[^ ^160^, ^161^ ^]^ Peng et al.^[^ ^48^ ^]^ prepared a P3‐K_0.5_MnO_2_ cathode consisting of hollow submicrospheres, which exhibited superior cycling stability to the bulk‐like P3‐K_0.5_MnO_2_ cathode.^[^ ^47^ ^]^ Weng et al. synthesized hierarchical K_0.7_Mn_0.7_Mg_0.3_O_2_ microparticles that demonstrated long cycling stability with 82.5% capacity retention after 400 cycles at 100 mA g^−1^ and fast rate capability.^[^ ^93^ ^]^ Deng et al. used microspheres to develop a P3‐K_0.5_Mn_0.72_Ni_0.15_Co_0.13_O_2_ cathode with a high tap density (1.91 g cm^−3^), which may have applications in high energy density KIBs.^[^ ^98^ ^]^ Morphological investigations revealed the presence of densely packed secondary microspheres consisting of submicron‐sized primary particles. The unique hierarchical structure facilitates rapid K^+^ transport owing to the short diffusion pathway and buffers the high stress resulting from continuous K^+^ deintercalation/intercalation. Wang et al.^[^ ^78^ ^]^ prepared interconnected K_0.7_Fe_0.5_Mn_0.5_O_2_ nanowires via an electrospinning method. The 3D framework with uniform carbon coating provided rapid K‐ion diffusion channels and a 3D electron transport network during K^+^ extraction/insertion. Recently, Hao et al.^[^ ^162^ ^]^ designed a microscale yolk–shell P3‐K_0.5_[Mn_0.85_Ni_0.1_Co_0.05_]O_2_ (YS‐KMNC) cathode which delivered a discharge capacity of 96 mAh g^−1^ at 20 mA g^−1^ and showed excellent cyclability with 80.5% capacity retention over 400 cycles at 200 mA g^−1^. Analysis of the strain variation during volume expansion of the cathode materials using finite element analysis (Figure 15c,d) revealed that the yolk–shell structured KMNC experienced lower internal stress than conventional KMNC. The conventional KMNC suffered with severe structural damage (Figure 15e,f), whereas the YS‐KMNC displayed enhanced cycling stability owing to its superior mechanical stability. These studies indicate that modification of the morphology of electrode materials can improve their conductivity and structural integrity, which enhance the durability and performance of batteries. Importantly, straightforward design strategies will facilitate the development of materials with the tunable morphology and high tap density required for practical applications.

### 5.7 Surface Modification

Surface coating can effectively protect layered materials from ambient air damage. Further, the coating can minimize parasitic reactions between the electrolyte and electrode surface and thus enhance the performance of electrode materials. Yu et al.^[^ ^163^ ^]^ reported that carbon coated on a NaCrO_2_ cathode suppressed moisture uptake owing to its hydrophobic properties and delayed exothermic decomposition of the cathode by preventing oxygen loss from the crystal lattice. Further, the coating improved the conductivity of the cathode, resulting in excellent capacity retention and superlative rate capability up to 150 C. The heteroatom (N, S)‐doped carbon coating on the Li[Ni_0.8_Co_0.1_Mn_0.1_]O_2_ cathode protects the electrode surface from HF acid generated by anionic oxidation of ${\mathrm{PF}}_{6}^{-}$ and thus enhances electrochemical performance.^[^ ^164^, ^165^ ^]^ Jo et al. reported that the electrical conductivity of K_2_V_3_O_8_ modified with carbon black was significantly better than that of unmodified K_2_V_3_O_8_.^[^ ^166^ ^]^ The K_2_V_3_O_8_/carbon cathode delivered a discharge capacity of 75 mAh g‐1 and high cyclability for 200 cycles with 80% capacity retention. Zhao et al.^[^ ^54^ ^]^ improved the stability of K_1.39_Mn_3_O_6_ microspheres by coating their surfaces with AlF_3_. The AlF_3_‐coated K_1.39_Mn_3_O_6_ exhibited superior reversible capacity and stability to those of the uncoated cathode (Figure 4b,c). This reveals that the AlF_3_ nanolayer coated on the surface of K_1.39_Mn_3_O_6_ enhances structural stability by buffering the structural changes during K^+^ extraction/insertion. In addition to surface coating, Lei et al.^[^ ^53^ ^]^ developed a new strategy involving the formation of a dual interface containing a K‐poor spinel interlayer and a stable solid‐electrolyte interface (SEI) film (Figure 4a). The dual interphase layers can accommodate the Jahn‐Teller distortion, mitigate Mn loss, and enhances K^+^ diffusion for redox reactions. Reports on this type of surface modification in K^+^‐layered materials are rare and thus further efforts are essential to realize efficient cathode materials. Though the surface coating of electrodes with conductive or inactive materials can improve their performance, an excess of these coating materials can also reduce the energy density of batteries.^[^ ^163^, ^164^ ^]^ Therefore, optimizing both the coating material and thickness is critical to obtain the desired battery performance.

### 5.8 Composite Structure Design

Composite electrode materials combining two different phase structures may demonstrate more interesting properties compared to their monophasic counterparts. The P2‐type materials used in Na^+^‐layered cathodes exhibit high structural stability and high‐rate capability owing to large Na^+^ diffusion channels; however, they suffer from Na^+^ deficiency. Conversely, O3‐type materials have higher Na^+^ content but display poor reversibility and rate performance.^[^ ^134^, ^167^ ^]^ Integrating these two different phase structures may give rise to synergistic effects, resulting in a composite material with the advantages of both monophasic materials. Chen et al.^[^ ^168^ ^]^ prepared P3/P2‐type biphasic layered Na_0.66_Co_0.5_Mn_0.5_O_2_ using sol–gel assisted heat treatment. Rietveld XRD patterns of the material revealed that the P3 phase (76.05%) was stronger than the P2 phase (23.95%). The prepared cathode demonstrated high cyclability even at high rates, taking advantage of the structural flexibility of the P3/P2 composite material. Qi et al.^[^ ^169^ ^]^ synthesized a series of O3/P2 Na*_x_* [Ni_0.2_Fe*_x_*_‐0.4_Mn_1.2_ *_x_*]O_2_ (*x* = 0.7–1.0) composites by tuning the sodium content. The O3/P2 ratio of the composite was optimized to achieve a balance among capacity, cycling stability, and rate capability. Liu et al.^[^ ^94^ ^]^ synthesized a KIB cathode, K_0.67‐_ *_x_* Na*_x_* Ni_0.17_Co_0.17_Mn_0.66_O_2_ (*x* ≤ 0.5), and found that varying the Na^+^ content altered the crystalline structure from a monophasic to a biphasic structure. The optimized P3/P2‐K_0.37_Na_0.3_Ni_0.17_Co_0.17_Mn_0.66_O_2_ cathode delivered a reversible capacity of 86.1 mAh g^−1^ at 20 mA g^−1^ and demonstrated superior cycling stability to the pristine P3‐K_0.67_Ni_0.17_Co_0.17_Mn_0.66_O_2_ over 100 cycles. However, excess Na^+^ hinders K^+^ diffusion and thus the dopant amount must be optimized to ensure optimal cathode performance. The design of multiphasic K^+^‐layered materials is in its infancy, and further research is required to understand the formation mechanism and the contribution of each phase to the stability of the crystal structure and the electrochemical performance.

### 5.9 Electrolyte Optimization

The electrolyte can affect many electrochemical parameters such as the reversible capacity, coulombic efficiency, rate performance, operating voltage, and energy density, and is, therefore, an important battery component. Accordingly, suitable electrolyte systems for application in practical potassium ion batteries must be explored. KPF_6_ dissolved in an ethylene carbonate/diethyl carbonate electrolyte is typically used in KIBs with layered cathodes (Table 1).^[^ ^46^, ^47^, ^48^, ^81^, ^82^, ^83^ ^]^ Yang et al. employed K_2_V_3_O_8_ as a high voltage cathode in a wide potential window of 1.5–4.5 V versus K/K^+^ using 7 m KFSI in ethylene carbonate/diethyl carbonate electrolyte.^[^ ^73^ ^]^ Lei et al. reported that a 6.0 m KFSI/diglyme (G2) electrolyte showed more stable SEI formation in P2‐K_0.67_MnO_2_ than carbonate‐based electrolytes.^[^ ^53^ ^]^ Masese et al. evaluated a K_0.4_Fe_0.5_Mn_0.5_O_2_ electrode in half‐cell with 0.5 m KTFSA in Pyr_13_TFSA and 1 m KFSA in Pyr_13_FSA ionic liquid electrolytes.^[^ ^80^ ^]^ The KFSA‐based ionic liquid electrolyte exhibits a lower viscosity and higher ionic conductivity than the KTFSA‐based ionic liquid electrolyte. Another study found that a P2‐K_2/3_Ni_1/3_Co_1/3_Te_1/3_O_2_ electrode with a 0.5 m KTFSI in Pyr_13_TFSI ionic liquid electrolyte exhibited a maximum average voltage of 4.3 V supported by an inductive [TeO_6_]^6 −^ framework. Interestingly, the presence of a fluoroethylene carbonate (FEC) additive in the electrolyte improved the performance of LIB and NIB systems.^[^ ^9^ ^]^ Bei et al. tested a K_1.64_Fe[FeII(CN)_6_]_0.89_·0.15H_2_O cathode in 0.7 m KPF_6_ in EC/DEC electrolyte with FEC (2 vol%). The FEC additive inhibited side reactions at the electrolyte and improved its coulombic efficiency.^[^ ^170^ ^]^ However, the FEC additive also formed a passivation layer on the graphite anode and thereby inhibited K^+^ ion transport, indicating that FEC is incompatible with graphite anodes. Park et al.^[^ ^171^ ^]^ investigated the influence of FEC concentration (1–5%) and found that K^+^ ion transport is inhibited by the SEI even at low FEC concentrations. Furthermore, other carbon anode materials such as mesocarbon microbeads (MCMB), graphene oxide, and sugar‐based hard carbon also exhibited similar issues in electrolytes containing FEC. These results indicate that K^+^‐impermeability may be an intrinsic property of FEC‐based electrolytes and further investigation is needed to fully understand the mechanism. The development of stable electrolyte systems with an appropriate salt, solvent, and additive is essential for achieving high‐performance KIBs. The design of efficient electrolyte systems must take into account several factors, including low viscosity, high ionic conductivity, high voltage operation, high‐temperature stability, and safety.

### 5.10 Full Cell Assembly

The development of practical KIBs requires the evaluation of electrode materials in a full cell assembly. Many K^+^‐layered oxide cathodes have been tested in half cell with a K‐metal anode (Table 1); however, the performance of these cathodes in a full cell has received comparatively little attention (Table 2). The use of K‐metal anode is not recommended in practical KIBs owing to the formation of dendrites on the metal surface, which may cause an internal short circuit and therefore carries safety risks.^[^ ^20^ ^]^ Therefore, testing of the electrode materials in a full cell with a K‐metal‐free anode is crucial because the performance of the cathode in a half cell may differ greatly from that in a full cell configuration. Vaalma et al.^[^ ^45^ ^]^ constructed a KIB full cell with a layered K_0.3_MnO_2_ cathode, hard carbon/carbon black composite anode, and 1.5 m KFSI in EC:DMC electrolyte. Before assembling the full cell, the hard carbon anode was prepotassiated to minimize the irreversible capacity arising from the formation of an SEI during the initial cycles. The KIB cell operating between 0.5 and 3.4 V delivered a discharge capacity of 92 mAh g^−1^ at 32 mA g^−1^ and exhibited 50% capacity retention after 100 cycles. Peng et al.^[^ ^48^ ^]^ synthesized a P3‐K_0.5_MnO_2_ cathode consisting of hollow submicrospheres and assembled a full cell paring with a graphite anode. The full cell displayed a discharge capacity of 60.2 mAh g^−1^ with a coulombic efficiency (CE) of 86.7% and reasonable rate performance. The K_0.6_CoO_2_/graphite full cell delivered a specific discharge capacity of ≈53 mAh g^−1^ at 3 mA g^−1^, but showed poor cycling efficiency owing to electrolyte consumption during the initial cycles.^[^ ^56^ ^]^ Deng et al. improved the performance of an P2‐K_0.6_CoO_2_ by synthesizing spherical microparticles that inhibited parasitic reactions and thus achieved high coulombic efficiency.^[^ ^57^ ^]^ The P2‐K_0.6_CoO_2_ microsphere//hard carbon full cell delivered a capacity of 71 mAh g^−1^ at 30 mAh g^−1^ and showed high cycling stability with 80% capacity retention over 100 cycles. The working potential and energy density of a full cell primarily depend on the performance of the cathode materials and thus it is imperative to employ rational material design to improve the overall performance of the cell. Since nonstoichiometric compounds often exhibit reduced capacity, carbon‐based anodes are typically prepotassiated for full cell assembly to compensate for any K^+^ deficiency.^[^ ^48^ ^]^ Kim et al. fabricated a full cell with a stoichiometric O3‐KCrO_2_ cathode and graphite anode, which delivered a discharge capacity of 97 mAh g^−1^ at 5 mA g^−1^.^[^ ^40^ ^]^ Unfortunately, this full cell demonstrated poor cyclability with only 50% capacity retention after just 10 cycles. Further efforts are necessary to improve the performance of such cells using suitable electrolytes and electrode materials.

**Figure 16:**
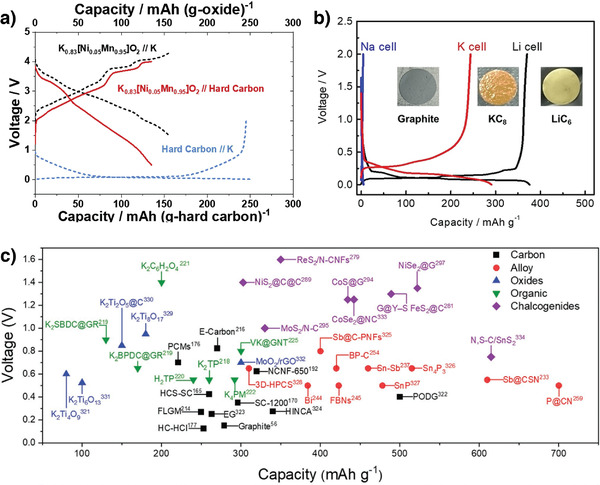
a) First charge/discharge profiles of full cells at 52 mA g^−1^. Reproduced with permission.^[^ ^88^ ^]^ Copyright 2020, Elsevier. b) Charge–discharge profiles of a graphite electrode in a Li cell with 1 m LiPF_6_/EC:DMC (black line), Na cell with NaPF_6_/EC:DEC (blue line) and in K cell with 1 m KFSI/EC:DEC (red line). Reproduced with permission.^[^ ^20^ ^]^ Copyright 2018, Wiley. c) Discharge capacity and average charge/discharge voltage of selected anode materials employed in potassium‐ion batteries. Reproduced with permission.^[^ ^172^ ^]^ Copyright 2021, Wiley.

Full cells with binary and ternary layered metal oxide cathodes exhibit superior performances to those with single‐metal oxide cathodes. Deng et al.^[^ ^44^ ^]^ constructed a P2‐ K_0.65_Fe_0.5_Mn_0.5_O_2_ microspheres//hard carbon full cell, which displayed a discharge capacity of 75 mAh g^−1^ at 100 mA g^−1^ and 80% capacity retention after 100 cycles. Wang et al.^[^ ^78^ ^]^ fabricated interconnected K_0.7_Fe_0.5_Mn_0.5_O_2_ nanowires by an electrospinning technique. The KIB full cell was assembled with a K_0.7_Fe_0.5_Mn_0.5_O_2_ cathode, soft carbon anode, and 0.8 m KPF_6_ in EC:DEC electrolyte. The K_0.7_Fe_0.5_Mn_0.5_O_2_//soft carbon cell delivered a capacity of 82 mAh g^−1^ with a high coulombic efficiency of 92% and retained 76% of its initial capacity over 250 cycles. Prior to constructing a full cell, carbon‐based anodes must be precycled in a half cell with a K‐metal counter electrode to avoid capacity loss during the initial cycles in the full cell. Further, the anode:cathode mass ratio must be balanced to ensure a capacity match between the electrodes. The full cell constructed with a P3‐K_0.54_[Co_0.5_Mn_0.5_]O_2_ cathode and hard carbon anode displayed a capacity of 96 mAh g^−1^ and retained 86% of its initial capacity over 100 cycles.^[^ ^82^ ^]^ Choi et al. assembled a full cell with a P’2‐K_0.83_[Ni_0.05_Mn_0.95_]O_2_ cathode and hard carbon anode, which exhibited an excellent capacity of 135 mAh g^−1^ at 52 mA g^−1^ and an energy density of 283 Wh kg^−1^ (**Figure** **16**a). Further, the cell showed long‐term cyclability with 80% capacity retention over 300 cycles.^[^ ^88^ ^]^ The P2‐K_0.44_Ni_0.22_Mn_0.78_O_2_//soft carbon full cell exhibited a discharge capacity of 70 mAh g^−1^ and maximum capacity retention of 90% after 500 cycles; however, it showed a low coulombic efficiency of 87.7% owing to SEI formation during the initial cycles, which gradually improved to 96.4% after activation of electrodes in 50 cycles.^[^ ^89^ ^]^ The ternary metal layered cathode P2‐K_0.75_[Mn_0.8_Ni_0.1_Fe_0.1_]O_2_ paired with a hard carbon anode exhibited a discharge capacity of 60 mAh g^−1^ at 20 mA g^−1^ and excellent long‐term stability with 60% capacity retention over 1000 continuous cycles.^[^ ^100^ ^]^ The performance of existing cathode materials is not sufficient to meet the requirements of practical KIBs, and thus further improvement in the capacity, operating voltage, structural stability, and air stability of these materials is vital for realizing practical KIBs.

The anode material is an important component of a full cell that determines the power density and safety of the batteries. Anode materials with high capacity and low operating potential are preferred for high energy density batteries. Although the K‐metal anode offers the highest energy density, its high reactivity and dendrite‐related safety issues limit its usage in practical batteries.^[^ ^19^ ^]^ Alternative anode materials including graphite and other carbon‐based materials, alloys, organic, conversion, intercalation, and polyanionic compounds have therefore been examined for application in KIBs. Anode materials are typically tested in a half‐cell containing a K‐metal counter electrode, which should be handled with utmost care owing to its high reactivity. Graphite anodes are typically used in commercial LIBs owing to their low operating potential and high gravimetric capacity. Unlike Na^+^ ions K^+^ ions can reversibly intercalate into graphite, which has garnered graphite electrodes much attention owing to their potential suitability for use in KIBs. The alkali‐metal intercalation behavior of graphite in Li, Na and K cells is depicted in Figure 16b. The graphite anode in a Li cell delivered a capacity of 370 mAh g^−1^, forming LiC_6_. In a K cell, the graphite anode forms KC_8_, giving a reversible capacity of 244 mAh g^−1^ which represents 87% of the cell's theoretical capacity. However, negligible Na^+^ insertion into the graphite was observed in the Na cell.^[^ ^15^ ^]^ Interestingly, K^+^ ions intercalate into graphite at a potential higher than the plating potential of K‐metal, which should ensure battery safety; however, the expansion of graphite upon significant K^+^ intercalation/deintercalation can cause the formation of an unstable SEI, resulting in poor coulombic efficiency.^[^ ^56^ ^]^ Komaba et al. demonstrated that the performance of the graphite anode can be improved by employing a suitable electrolyte and binder. Graphite used with sodium carboxymethyl cellulose binder shows a coulombic efficiency of 89%, greater than those of sodium polyacrylate (CE = 79%) and polyvinylidene fluoride (CE = 59%) binders.^[^ ^15^ ^]^ In addition to graphite, several carbonaceous materials such as hard, soft, porous, and heteroatom‐doped carbon have been employed in K^+^ storage studies. These types of carbon material show superior capacity and cyclic stability than graphitic carbon; however, they also exhibit low coulombic efficiency and high overpotential, resulting in low‐energy‐density batteries.^[^ ^172^, ^173^ ^]^ The discharge capacities and average voltage of various anode materials used in KIBs are shown in Figure 16c.

Recently, alloy‐type anode materials such as Si, Sb, Sn, and P have been studied in KIBs because they can deliver higher capacity than intercalation‐type carbon materials. However, these alloy anodes suffer from severe volume changes, pulverization of the active materials, low conductivity, and sluggish reaction kinetics.^[^ ^174^ ^]^ Various strategies have been developed to address these issues, including the design of nanostructured alloys, composite alloys with carbon and metal oxides, and optimizing the composition and amount of electrolyte and additives. Conversion‐type anodes such as metal sulfides and metal oxides have been tested; however, many challenges must be resolved before this type of anode material can be employed in practical applications. Conversion‐type anodes exhibit high polarization voltages and volume changes, with low coulombic efficiency, and slow reaction kinetics.^[^ ^21^, ^172^ ^]^ Organic anode materials (K_2_TP, K_2_PC) offer a tunable redox potential, structural flexibility, and a greater number of voids to facilitate K^+^ diffusion. Moreover, the discharge profile of organic anodes occurs at a potential higher than the potassium plating potential, which improves the safety of the full cell. However, the coulombic efficiency, capacity, and rate capability of organic anode materials must be improved to realize a practical KIB full cell.^[^ ^21^, ^32^, ^33^, ^172^ ^]^

In summary, the performance of a full cell is dependent on the synergistic effect between the anode, cathode, and electrolyte components. Practical batteries require safe electrode materials with high capacity, high operating voltage, long cycle life, high charge/discharge rate, and structural stability. Anode materials possessing a high capacity, low operating potential, and high coulombic efficiency are suitable for use in full cells. The performance of carbon‐based anode materials can be enhanced by doping with heteroatoms tailoring their morphologies using nanostructures. The K‐metal anode is used as counter/reference electrode in half‐cell studies; however, the highly reactive metal surface can undergo side reactions with the electrolyte, causing contact issues. The performance of electrode materials in a half cell differs from that in a full cell configuration owing to the unstable reference electrode in the half cell, and thus a standard reference electrode is essential for half‐cell testing. The energy density of a battery is determined by the cathode performance and therefore the development of novel cathode materials is vital. Further, the design of KIBs for practical applications must consider the capacity balance between anode and cathode, stable electrolyte salts, solvents, binders, operating voltage range, cost and safety to ensure that KIBs reach their full potential.

The metal elements used in the design of K^+^‐based layered cathodes for application in KIBs are summarized in **Table** **3**. The single transition‐metal oxide compounds that crystalize in layered structure have been developed using Mn, Co, Cr, and V metals. The layered single‐metal oxide cathodes could deliver reasonable capacity; however, these compounds suffer from multiple phase transitions, stepwise voltage, K^+^/vacancy ordering, slope voltage, capacity fading, and structural instability. Moreover, the expensive and toxic nature of Co, Cr, and V‐based layered compounds make them unfavorable for practical applications. Conversely, the low‐cost and nontoxic Mn‐based layered compounds are considered as promising candidates, however, the Mn^3+^ associated Jahn‐Teller distortions need to be addressed.^[^ ^40^, ^46^, ^57^, ^73^ ^]^ To circumvent the issues related with layered single metal compounds, many efforts have been focused on developing binary metal or multimetal compounds by introducing two or more metals into the single metal oxide systems. Various binary metal oxide compounds have been developed by using electrochemically active metals (Ni, Fe, Co, Mn) and inactive metal (Ti, Mg, Al, Sb, Te). The metal substitution strategy combines synergistic contributions from different metals leading to improved structural and electrochemical properties. For instance, Ni^2+^ substitution improved structural stability by alleviating J‐T distortions and the Ni^2+^/^4+^ redox couple provided high operating voltage.^[^ ^84^ ^]^ The Fe^3+^ doping is able to provide desirable capacity involving high voltage redox couple (Fe^3+/4+^).^[^ ^44^ ^]^ The Fe/Mn‐based compounds are suitable for practical KIBs because both the elements (Fe, Mn) are low‐cost, abundant, and environmentally benign. The Ti^4+^ substation increased the valence state of Mn^3+^ that suppresses the J‐T distortions. Further, the electrochemical inactive Ti^4+^ prevents the layer gliding and thus improving structural stability during K^+^ deintercalation/intercalation.^[^ ^103^ ^]^ Generally, the doping of electrochemically inactive metal reduces the specific capacity and thus the doping amount must be adjusted to obtain enhanced capacity and stability. The doping metals with high electronegativity (Sb^5+^, Te^6+^) could provide high operating voltage due to the inductive effect.^[^ ^12^, ^106^ ^]^ In a word, developing K^+^‐layered oxide materials with suitable elements and compositions could possibly improve structural stability, specific capacity, average operating voltage, and cycling stability.

**Table 3:** Summary of metal elements characteristics and functions in K^+^‐layered oxide cathodes

Element	Elemental abundance [ppm]^[^ ^60^ ^]^	Cathode material	Redox couple	Advantage	Disadvantage	Voltage window [V]	Discharge capacity [mAh g^−1^]/current density [mA g^−1^]	Refs.
Mn	950	P3‐K_0.45_MnO_2_	Mn^3+/4+^	Low‐cost, high capacity	J‐T effect active Mn^3+^	1.5–4.0	128.6/20	[46]
Co	25	P2‐K_0.6_CoO_2_	Co^3+/4+^	Good ion kinetics	Expensive, toxic	1.7–4.0	82/10	[57]
Cr	102	O3‐KCrO_2_	Cr^3+/4+^	High capacity	Toxic, complex phase transitions	1.5–4.0	92/5	[40]
V	120	K_2_V_3_O_8_	V^4+/5+^	High voltage	Toxic, expensive	1.5–4.5	107.8/10	[73]
Ni	84	P2‐K_0.75_[Ni_1/3_Mn_2/3_]O_2_	Ni^2+/4+^, Mn^4+^‐inactive	High voltage, suppress J‐T effect	Expensive	1.5–4.3	110/20	[84]
Fe	56300	P2‐K_0.65_Fe_0.5_Mn_0.5_O_2_	Fe^3+/4+^, Mn^3+/4+^	Low‐cost, high voltage	J‐T active Fe^4+^	1.5–4.2	151/20	[44]
Ti	5600	K_0.4_Fe_0.1_Mn_0.8_Ti_0.1_O_2_	Mn^3+/4+^, Fe^3+/4+^, Ti^4+^‐inactive	Increase ionicity, structural stability	Inactive Ti^4+^ reduce capacity	1.5–4.0	117/10	[103]
Mg	23300	K_0.7_Mn_0.7_Mg_0.3_O_2_	Mn^3+/4+^, Mg^2+^‐inactive	Suppress phase changes	Inactive Mg^2+^ reduce capacity	1.5–4.0	144.5/20	[93]
Al	82300	K_0.45_Ni_0.1_Co_0.1_Al_0.05_Mn_0.75_O_2_	Mn^3+/4+^, Al^3+^‐inactive	Suppress J‐T distortions	Capacity loss	1.5–4.0	84.5/20	[99]
Sb	0.2	P2‐K_0.7_[Cr_0.85_Sb_0.15_]O_2_	Cr^3+/4+^, Sb^5+^‐inactive	High voltage	Capacity loss	1.5–4.3	70/15.4	[12]
Te	0.001	P2‐K_2/3_Ni_2/3_Te_1/3_O_2_	Ni^2+/4+^, Te^6+^‐inactive	High voltage	Capacity loss	1.3–4.7	65/6.4	[106]

## 6 Summary and Future Perspectives

The potassium‐ion batteries (KIBs) are emerging as promising complements to lithium‐ion batteries (LIBs), because of the abundant potassium resources, low standard reduction potential of potassium, and reversible electrochemistry in graphite anode. However, it is imperative to develop cathode materials with high capacity and structural stability for realizing practical KIBs. In this review, recent developments, classifications, electrochemical performances, and structural changes of layered metal‐oxide cathodes have been discussed in detail for application to KIBs. Despite several KIB achievements, however, many challenges remain and must be overcome for commercial KIB applications. A major drawback is that most K^+^‐ion layered metal oxide cathodes are K^+^‐ion deficient compounds, which limit the KIB practical capacity because strong K^+^–K^+^ interactions in stoichiometric K^+^‐ion layered oxides destabilize the layered structure and form K^+^‐ion‐deficient compounds or other 3D structures. In half‐cell configurations, the initial potassium loss related to the solid electrolyte interface (SEI) formation is compensated by the potassium metal anode. However, a full‐cell KIB constructed using a carbon‐based anode and a K^+^‐deficient layered cathode demonstrates low capacity because the cathode is the K^+^‐ion reservoir. To overcome these issues, K^+^‐rich layered compounds can be synthesized by introducing suitable metal cations. Furthermore, prepotassiation, as used in LIBs, can also be employed in KIBs. K^+^‐ion layered oxide compounds demonstrate steeper voltage curves and more variation in stepwise voltage than their Li^+^ and Na^+^ counterparts mainly because strong K^+^–K^+^ repulsion causes K^+^/vacancy ordering as a function of K^+^‐ion content and transition‐metal charge ordering, thereby leading to numerous phase transitions and voltage steps. Further studies are required to design multimetal compounds with various redox‐active and ‐inactive metals to suppress K^+^/ordering and obtain smoother voltage profiles for K^+^‐ion layered oxide compounds.

K^+^‐layered metal oxide compounds exhibiting larger interlayer spacing easily enable H_2_O/CO_2_ intercalation in the ambient atmosphere, thereby causing structural damage and poor electrochemical activity. Thus, most layered compounds must be handled in an inert atmosphere, which increases manufacturing and transportation costs. Therefore, designing and developing air‐stable layered metal oxide compounds are critical for fabricating sustainable KIBs. Taking cues from LIBs and NIBs, research efforts must focus on developing air‐stable K^+^‐ion layered metal oxides by following different strategies such as cation substitutions, designing specific cathode compositions, and applying protective surface coatings. Nevertheless, Mn‐based layered compounds appear to be promising materials for application to KIB cathodes because of their low cost and eco‐friendliness. Moreover, Mn^3+^‐induced J‐T‐distortion, structural deterioration must be suppressed by cation doping to optimize the cathode electrochemical performance. In addition to designing structures, electrode micro/nanomaterials can be fabricated to exhibit porous morphologies and mitigate volumetric changes and facilitate ion/electron transport during battery operation. Furthermore, extensive efforts must focus on investigating KIB full cells because they are the foundation for fabricating commercial KIBs exhibiting satisfactory electrochemical performance. Another KIB challenge is exploring electrolyte salts, solvents, and additives. Electrolytes must be compatible with KIB anode and cathode materials while avoiding aluminum current‐collector corrosion and electrolyte decomposition at upper cutoff voltages. Furthermore, although fluoroethylene carbonate (FEC) forms a stable SEI in LIBs and NIBs, it is incompatible with KIB graphite anodes; thus, systematic investigations are required to find suitable KIB electrolyte additives. Despite many breakthroughs, KIBs remain in their infancy, leaving many challenges and opportunities to be addressed by the research fraternity and industrialists to scale KIBs from the laboratory to practical industrial applications.

## Conflict of Interest

The authors declare no conflict of interest.
